# Mental Health Outcomes After Neurocritical Care: A Systematic Review and Meta-analysis

**DOI:** 10.1007/s12028-026-02576-2

**Published:** 2026-07-06

**Authors:** Jamie Nicole LaBuzetta, Henry G. Chen, Nicholas Ibrahim, Onyekachi Ezeokeke, Sanil Gandhi, Atul Malhotra, Victor D. Dinglas, Dale M. Needham, Biren B. Kamdar

**Affiliations:** 1 Department of Neurosciences, Division of Neurocritical Care, UC San Diego Health, University of California - San Diego, La Jolla, CA, USA.; 2 School of Medicine, University of California - San Diego, La Jolla, CA, USA.; 3 Department of Medicine, UC Irvine School of Medicine, University of California - Irvine, Irvine, CA, USA.; 4 Division of Pulmonary and Critical Care Medicine, School of Medicine, Johns Hopkins University, Baltimore, MD, USA.; 5 Outcomes After Critical Illness and Surgery (OACIS) Research Group, Johns Hopkins University, Baltimore, MD, USA.; 6 Department of Physical Medicine and Rehabilitation, School of Medicine, Johns Hopkins University, Baltimore, MD, USA.; 7 Department of Medicine, Division of Pulmonary, Critical Care and Sleep Medicine, UC San Diego Health, University of California - San Diego, La Jolla, CA, USA.; 8 Department of Medicine, VA San Diego Healthcare System, La Jolla, CA, USA.

**Keywords:** Mental health, Depression, Anxiety, Post-traumatic stress, Critical care, Neurology, Neurocritical

## Abstract

**Objective:**

In this study, we sought to conduct a systematic review and meta-analysis of mental health outcomes in survivors of neurocritical illness.

**Methods:**

Literature databases [PubMed, Embase, the Cumulative Index to Nursing and Allied Health Literature (CINAHL), and PsycInfo] were searched for terms relating to critical illness, intensive care, and outcomes from January 1970 to June 2024. English-language studies of adults with critically illness with a primary neurological diagnosis were included if they reported on mental health outcomes [specifically, depression, anxiety, post-traumatic stress (PTS), or general mental health]. Data extraction was performed, in duplicate, for prespecified variables related to study outcomes. Random effects meta-analyses were conducted to estimate pooled prevalence and symptom severity.

**Results:**

Of more than 33,000 abstracts screened, 24 publications reported on mental health outcomes: 19 reported on depression outcomes, 11 on anxiety, 7 on PTS, and 8 on general mental health. The median [interquartile range (IQR)] time to first depression, anxiety, and/or PTS assessment was 3 (1.75, 12), 4.5 (1.1, 7.5), and 3 (0, 3) months, respectively. The most common assessment tools were the Hospital Anxiety and Depression Scale, Depression Subscale (HADS-D; *n* = 8) and the Hospital Anxiety and Depression Scale, Anxiety Subscale HADS-A (*n* = 8), and Post-traumatic Stress Disorder Checklist for the Diagnostic and Statistical Manual of Mental Disorders, Fifth Edition (PCL-5)/Post-traumatic Stress Disorder Checklist, Civilian Version (PCL-C) for PTS (*n* = 4). General mental health outcomes were studied using seven unique tools at a median (IQR) follow-up time of 3 (0.5, 6) months. Pooled depression prevalence [95% confidence interval (CI)] was 24% (20–29%) among publications using HADS-D and 26% (16–38%) in publications using any assessment tool. Pooled anxiety prevalence was 37% (21–56%) using HADS-A and was 32% (18–51%) using any assessment tool. PTS prevalence was 14% (8–21%). Heterogeneity of assessment tools precluded a pooled analysis of general mental health.

**Conclusions:**

These findings highlight the burden of mental health symptoms following neurocritical care illness, with prevalences higher than the general population. These findings were impacted by substantial between-study heterogeneity—particularly in assessment tools and timing of evaluations—limiting precise prevalence estimation.

## Introduction

Surviving critical illness marks the beginning of the intensive care unit (ICU) recovery process, a journey that may be complicated by new and persistent cognitive, physical, and mental health impairments. Collectively termed “post-intensive care syndrome” (PICS), this constellation of challenges can impact quality of life for both patients and their caregivers [1–6]. Mental health symptoms, including depression, anxiety, and post-traumatic stress, are among the most common PICS manifestations and may persist for months or years after hospital discharge.

While PICS-related mental health symptoms are well described in general ICU populations, their interpretation in survivors of neurocritical care is more complex [7–12]. Some symptoms may arise from the underlying neurological disease process (e.g., post-stroke depression), whereas others may reflect the experience of critical illness. Historically, PICS symptoms have been considered distinct from the sequelae observed in survivors after neurocritical care, as the acute structural neurological injury (“macro”-injury; e.g., hemorrhagic stroke) might obscure classical PICS symptoms [13]. As a result, PICS-related research has largely excluded the neurocritical care population. Nonetheless, PICS does not spare survivors of neurocritical illness, and increasing attention has focused on understanding the cognitive, physical, mental health, return-to-work, and quality-of-life outcomes in this population [14].

A recent scoping review summarized the literature surrounding nonmotor outcomes in survivors after neurocritical care [14]. To build upon this work and address remaining gaps, we performed a systematic review and meta-analysis of studies evaluating mental health outcomes in survivors after neurocritical care.

## Methods

### Study Design

This systematic review was conducted according to the Preferred Reporting Items for Systematic Reviews and Meta-Analyses (PRISMA; Supplementary Table S1) guidelines [15] and registered on International Prospective Register of Systematic Reviews (PROSPERO; ID = CRD42020169543). This report represents the synthesis and outcomes stage of a previously published protocol [14, 16].

### Research Question

Among studies published between 1970 and 2024, what are the prevalence and severity of depression, anxiety, and post-traumatic stress in survivors of acute neurological injury requiring intensive care?

### Identification of Eligible Studies

We searched PubMed, Embase, the Cumulative Index to Nursing and Allied Health Literature (CINAHL), and PsycInfo from 1 January 1970 to 30 June 2024 using previously published search terms finalized prior to database searching (Supplementary Table S2) [14, 17]. The strategy incorporated controlled vocabulary [e.g., Medical Subject Headings (MeSH) terms] and free-text keywords related to intensive care and post-ICU outcomes, combined using Boolean operators [14]. To maximize sensitivity, neurocritical care populations and mental health outcomes were applied during screening rather than the initial scoping search stage. The original search was performed in 2013 [17], with iterative refreshes in 2015, 2016, 2018, 2021, 2022, and 2024 yielding the totality of citations considered. A librarian with expertise in systematic reviews assisted with each search iteration.

### Study Selection

Citations were compiled, duplicates removed, and abstracts screened by a team of trained reviewers. Abstracts were screened in duplicate, with discrepancies resolved through consensus.

Full-text articles were obtained for abstracts meeting the following criteria: (1) original research in patients requiring ICU-level care, (2) English language, (3) adults aged ≥ 18 years, (4) acute neurological injury requiring ICU care (e.g., stroke, traumatic brain injury, subarachnoid hemorrhage, status epilepticus), and (5) reporting of mental health outcomes. Full texts were also obtained for relevant citations lacking abstracts.

During full-text review, the same inclusion criteria were applied. For mixed ICU cohorts, studies were included only if patients with neurological illness or injury were explicitly identified or if neurological-specific data could be obtained from the authors. When neurological data could not be separated, corresponding authors were contacted; studies were excluded if authors replied to say such data were unavailable (*n* = 3).

### Risk of Bias

Two independent reviewers (N.I., S.G.) assessed the risk of bias for each study using the Newcastle–Ottawa Scale [18]. Domains included exposure ascertainment, confirmation that outcomes were absent at study onset, outcome assessment, and adequacy of follow-up (Supplementary Table S7). Discrepancies were resolved through consensus.

### Data Abstraction and Summarization

Data abstraction was performed independently and in duplicate by two-person teams. For mixed cohorts, only the neurocritical-care-specific data were recorded. Discrepancies were resolved via consensus or, when necessary, adjudication by an independent team member (H.C., B.B.K., or J.N.L.).

Variables of interest were prespecified and entered into a standardized form in Microsoft Excel. Extracted variables included the author(s), publication year, country, study design, study location, sample size, patient demographics, and quantitative mental health outcomes. Primary outcomes were depression, anxiety, post-traumatic stress symptoms, and general mental health.

Descriptive statistics used to summarize data were generated using Microsoft Excel (version 16.89).

### Meta-analysis of Mental Health Outcomes

Random-effects meta-analyses were performed to estimate pooled prevalence and symptom severity of depression, anxiety, and post-traumatic stress among survivors after neurocritical care. Studies were grouped according to reported follow-up timepoints.

For prevalence analyses, log odds and corresponding standard errors were calculated from reported proportions and pooled using random-effects models. For depression and anxiety, primary prevalence analyses focused on studies using the Hospital Anxiety and Depression Scale (HADS), the most commonly reported instrument. Clinically significant symptoms were defined using validated HADS Depression Subscale (HADS-D) and HADS Anxiety Subscale (HADS-A) thresholds, with ≥ 8 demonstrating optimal sensitivity and specificity [19, 20]. When sufficient data were available, prevalence estimates were pooled within instrument-specific subsets (e.g., HADS-D). Secondary analyses combined prevalence estimates across validated instruments and measurement approaches. For post-traumatic stress symptoms, no single assessment tool predominated; therefore, pooled prevalence estimates were derived using study-level thresholds where available.

Sensitivity analyses were conducted to evaluate the robustness of pooled prevalence estimates to analytic decisions and study characteristics. These analyses included restricting analyses to validated instruments only, excluding outlier studies [defined as having a study prevalence confidence interval (CI) that fell outside the pooled prevalence confidence interval—a method previously used in meta-analyses [21, 22], excluding a large administrative dataset study, and grouping follow-up intervals into broader time windows (≤ 6 months and ≥ 12 months).

For severity analyses, continuous scores were pooled only when the same instrument was used across studies, specifically HADS-D and HADS-A for depression and anxiety, respectively. For post-traumatic stress symptoms, severity scores were pooled for studies using the Post-traumatic Stress Disorder Checklist (PCL), although these studies did not consistently report corresponding prevalence estimates. Where medians and interquartile ranges (IQR) were reported, means and standard deviations (SD) were estimated using established methods [23].

Similar to prior analyses [24, 25], meta-analyses were performed using the web-based platform MetaAnalysisOnline (https://metaanalysisonline.com) [26], applying the DerSimonian and Laird random-effects model with inverse variance weighting and logit transformation. The Knapp–Hartung adjustment was applied to improve confidence interval accuracy.

Forest plots were generated for each mental health domain, stratified by follow-up interval. Between-study heterogeneity was assessed using the *I*^2^ statistic, with ≥ 50% representing substantial heterogeneity [27]. Subgroup differences across follow-up timepoints were evaluated using Cochran’s *Q*-test, with *p* < 0.05 considered statistically significant. Insufficient studies were available within each outcome and timepoint (≤ 10 studies) to conduct meta-regression [28, 29] or to formally assess publication bias (e.g., funnel plots) [30], consistent with prior post-ICU depression and anxiety meta-analyses [19, 20].

### Institutional Review Board (IRB)

As a systematic review and meta-analysis of published data, this study did not involve human or animal subjects research and was exempt from institutional review board approval.

## Results

**Fig. 1:**
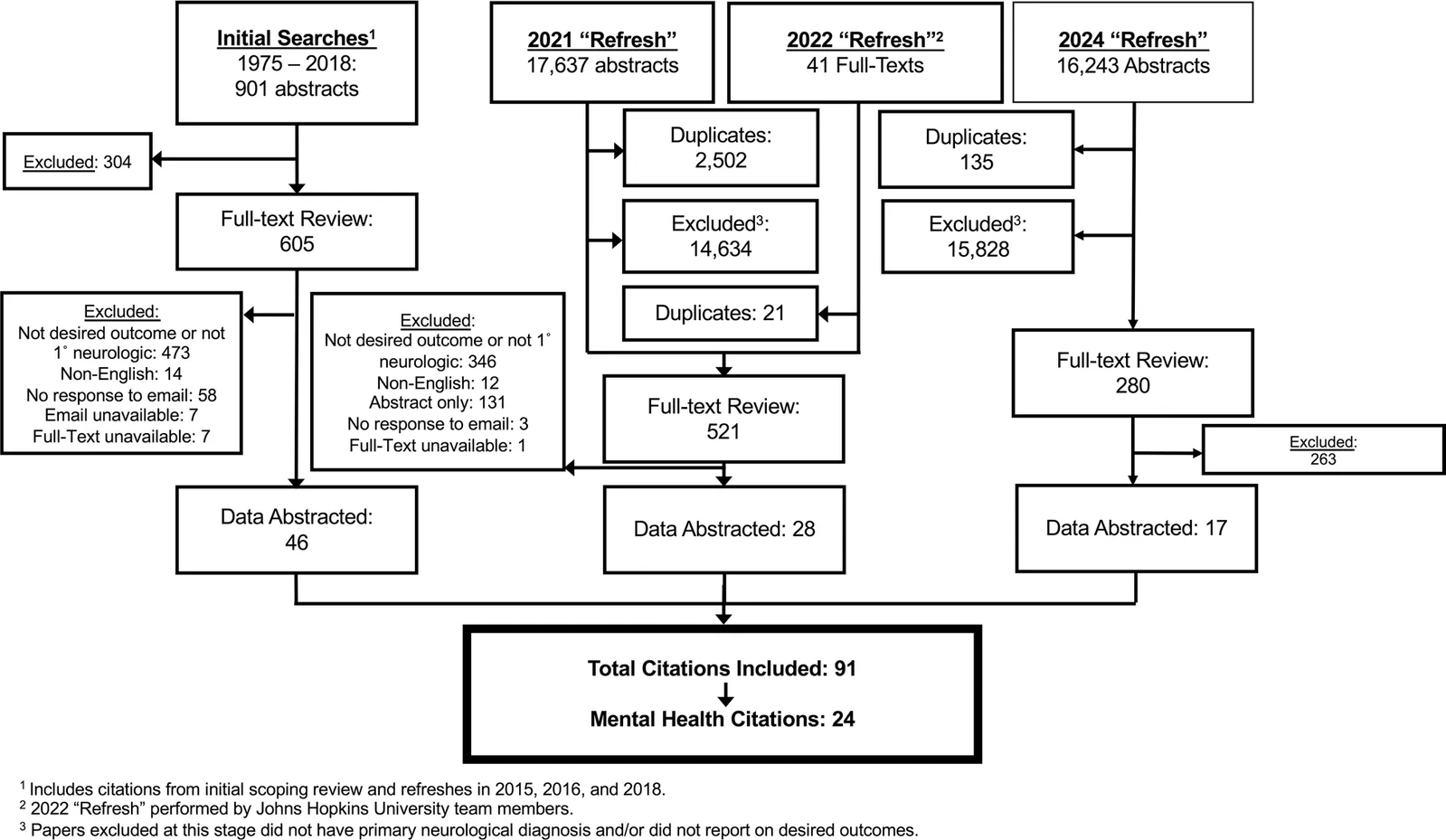
Systematic review flow diagram highlighting multiple searches and yielding 24 citations included in this systematic review

Our literature search yielded 16,243 citations. After removing duplicates, 16,108 abstracts were screened and 280 full texts reviewed, yielding 24 studies for inclusion (Fig. 1). Overall, 19 publications reported on depression [9, 31–48], 11 on anxiety [7, 9, 31–33, 35, 42, 43, 45, 46, 48, 49], 7 on post-traumatic stress symptoms [32, 33, 35, 42, 47, 50, 51], and 8 on general mental health outcomes [32, 35, 40, 41, 47, 49, 52, 53] (Table 1). One author group had three reports on different mental health outcomes using the same participant population [40, 41, 49], which we treated as a single cohort.

### Depression

Twelve unique measurement tools were used to assess depression, with the HADS-D scale being the most common (*n* = 8 studies; Supplementary Table S3). Measurements were performed at a median of 3 (IQR 1.75, 12; max = 152.5) months. Across all studies, 94,404 patients were evaluated, including one large retrospective national database cohort study [39] with 90,655 neurocritical care survivors. Among studies using HADS-D only, the pooled prevalence of depression symptoms was 24% (95% CI 20–29%; *I*^2^ = 0%), with low heterogeneity (Supplementary Fig. S1). When all measurement approaches were included, the pooled prevalence of depression symptoms was 25% (95% CI 16–37%, *I*^2^ = n/a) at 0–3 months, 31% (95% CI 10–64%, *I*^2^ = 92.8%) at 6 months, 16% (95% CI 2–62%, *I*^2^ = 96.3%) at 12 months, and 31% (95% CI 3–85%, *I*^2^ = 90.8%) at ≥ 18 months, with an overall prevalence of 26% (95% CI 16–38%) (Table 2) and significant heterogeneity (*I*^2^ = 99.6%) (Supplementary Fig. S3).

Sensitivity analyses demonstrated stable prevalence estimates of 24–29% across analytic approaches, including use of broader follow-up intervals of ≤ 6 months and ≥ 12 months (Table 2). Restricting analyses to studies using validated instruments yielded a pooled depression prevalence of 28% (95% CI 18–41%, *I*^2^ = 94%; Supplementary Fig. S3a–b). Excluding outlier studies resulted in a pooled prevalence of 25% (95% CI 17–34%, *I*^2^ = 94.5%; Supplementary Fig. S3c–d). One study [39] used a large administrative dataset that substantially exceeded the sample size of other studies. To evaluate its influence on pooled estimates, analyses repeated excluding this dataset resulted in a pooled depression prevalence of 29% (95% CI 20–40%, *I*^2^ = 93.1%; Supplementary Fig. S2c–d).

Depressive symptom severity was assessed in seven studies (*n* = 839), yielding a pooled mean HADS-D score of 5.72 (95% CI 4.69–6.75; *I*^2^ = 89.6%), indicating mild-to-moderate symptom burden (a HADS-D cutoff of ≥ 8 represents *possible* or *probable* clinical major depressive disorder [54]) (Supplementary Fig. S4). Among studies assessing the same follow-up timepoint using the HADS-D, mean (SD) severity scores ranged from 4.4 (4.4) [45] to 7.9 (4.1) [33] at discharge to 4.6 (4.0) [40] to 8.6 (2.9) [32] at 3 months (Supplementary Table S3).

### Anxiety

Four validated instruments were used to assess anxiety, with HADS-A being the most frequently employed (*n* = 8 studies; Supplementary Table S4). Prospective measurements were performed at a median of 4.5 (IQR 1.1, 7.5) months. Among only studies using HADS-A (*n* = 6 studies, *n* = 758 subjects), the pooled prevalence was 37% (95% CI 21–56%; *I*^2^ = 94%) (Table 2; Supplementary Fig. S5). Across all studies and tools, 3127 patient assessments were made. Overall anxiety symptom prevalence ranged from 13.2% [45] to 77.8% [32] (Supplementary Fig. S6). Pooled prevalence at specific timepoints using validated assessment methods was estimated at 25% (95% CI 0–100%; *I*^2^ = 96.6%) at 0 months, 32% (95% CI 4–85%; *I*^2^ = 95.4%) at 6 months, and 45% (95% CI 5–93%; *I*^2^ = 40.0%) at 12 months.

Sensitivity analyses demonstrated stable prevalence estimates of 32–43% across analytic approaches, including use of broader follow-up intervals of ≤ 6 months and ≥ 12 months (Table 2). Restricting analyses to studies using any validated measurement, pooled prevalence of anxiety was 32% (95% CI 18–51%, *I*^2^ = 96.6%; Supplementary Fig. S6a–b), but restricting analysis to validated studies without outliers resulted in a pooled anxiety prevalence of 43% (95% CI 35–52%, *I*^2^ = 32.0%; Supplementary Fig. S6c–d).

Severity of anxiety symptoms was assessed in seven studies (*n* = 885). At similar follow-up timepoints, mean (SD) anxiety severity ranged from 5.3 (4.4) [45] to 10.2 (4.9) [32] at discharge, 5.9 (4.7) [33] to 12.7 (5.9) [32] at 1.5 months, and 6.4 (5.7) [49] to 9.9 (6.5) [32] at 6 months. There was an overall pooled mean HADS-A score of 6.70 (95% CI 5.87–7.52; *I*^2^ = 82%) (Supplementary Fig. S7); a score of ≥ 8 indicates a possible clinical case of anxiety disorder [55].

### Post-traumatic Stress

Six studies (*n* = 2506) assessed post-traumatic stress symptoms using five different instruments, most commonly the Post-traumatic Stress Disorder Checklist for the Diagnostic and Statistical Manual of Mental Disorders, Fifth Edition (PCL-5), or the Post-traumatic Stress Disorder Checklist, Civilian Version (PCL-C), at a median of 3 (IQR 0, 3) months (Supplementary Table S5). The pooled prevalence of post-traumatic symptoms measured by validated tools was 14% (95% CI 8–21%; *I*^2^ = 79%) (Table 2; Supplementary Fig. S8).

Severity was assessed in two studies (*n* = 65); mean (SD) PCL scores ranged 30.5 (11.5) [33] to 43.8 (10.6) [32] at 1.5 months, and 30.3 (SD unavailable) [51] to 38.9 (11.8) [32] at 3 months. A pooled mean PCL-C score of 36.3 (95% CI 13.0–59.6; *I*^2^ = 20%) (Supplementary Fig. S9) is reported; a score ≥ 30 is consistent with moderate to moderately high severity of symptoms [56]. Unlike for depression and anxiety, heterogeneity in PTS severity scores was low but included a small number of studies (*n* = 2).

### General Mental Health

Seven unique measurement tools were used to evaluate general mental health in 408 participants at a median of 3 (IQR 0.5, 6) months following discharge. None of the measurement tools was duplicated between publications, and each measured slightly different aspects of mental health, such as resiliency, psychosis, coping, and perceived social support (Supplementary Table S6). These measured constructs are not equivalent to standard psychiatric outcomes, so post hoc pooling with depression, anxiety, or post-traumatic stress data was not attempted.

### Risk of Bias

The categories with least risk of bias pertained to ascertainment of exposure(s), follow-up timeframe such that outcome could occur, and assessment of outcome (Supplementary Table S7). However, many studies had risk of bias related to adequacy of follow-up. Of the 24 studies, 50% did not have adequate follow-up.

## Discussion

This systematic review and meta-analysis is the first, to our knowledge, to provide detailed analysis on mental health outcomes in survivors of acute neurological injury requiring ICU hospitalization. We identified 24 publications reporting on depression, anxiety, post-traumatic stress, and/or general mental health outcomes in survivors after neurocritical care.

Although studies were heterogenous in terms of their designs and study population, our results describe the high disease burden and severity of mental health outcomes in survivors after neurocritical care. The pooled prevalence of depression (24–29%), anxiety (32–43%), and post-traumatic stress (14%) in this analysis are above levels described in the general population [57–59]. In considering the severity of mental health challenges, it is important to understand stratification within the most common screening tools. A HADS-D score of ≥ 8 denotes the probable presence of major clinical depressive symptoms, with higher scores indicating more severe depression. Some studies included in this manuscript reported mean scores as high as 10.1, indicating severe impact [32], though it is also noted that the pooled severity scores have wide confidence intervals and significant heterogeneity. Further, a HADS-A score ≥ 8 denotes the presence of anxiety, again with higher scores indicating more severe anxiety; the highest mean HADS-A score reported within the reviewed manuscripts was 12.7 at 1.5 months of follow-up [33]. Again, pooled anxiety severity scores are plagued by significant heterogeneity. With regards to post-traumatic stress symptoms, a total score of ≥ 33 on the PCL is often considered a threshold for clinically significant post-traumatic stress symptoms; therefore, the pooled severity scores seen here have potential clinical relevance while also noting the small number of studies contributing to the pooled score of 36.3.

To address heterogeneity and variability in study design, we conducted comprehensive sensitivity analyses, combining prevalence across validated instruments only, including all measurement methods, grouping studies by broader follow-up intervals (≤ 6 months and ≥ 12 months), and excluding outlier studies. Across these analyses, pooled prevalence estimates remained consistent despite substantial heterogeneity, supporting their interpretation as indicators of overall burden rather than precise point estimates. Importantly, exclusion of outlier studies did not meaningfully alter pooled estimates. As a notable example, sensitivity analysis excluding the study by Liao and colleagues [39]—a large retrospective cohort that identified depression through administrative diagnostic codes rather than validated screening instruments—did not significantly alter the pooled prevalence estimates. This suggests that, despite its large sample size from Taiwan’s National Health Insurance database, the study by Liao et al. [39] did not disproportionately influence our results, further supporting the robustness of our findings.

Although this review focused on the neurocritical population, these results parallel those involving survivors of general critical illness. Specifically, our pooled depression prevalence of 28% using validated tools is consistent with reported long-term rates of depression among survivors of acute respiratory distress syndrome (30% at 12 months) [60], as well as consistent with a depression prevalence in survivors of general critical care of 29% at 12–14 months in a recent meta-analysis [19]. Published rates of anxiety often use the HADS-A [20] and reported pooled prevalence in the general critical care population ranges from 32% (at ≤ 3 months) to 40% (at 12–14 months) [20], which is similar to the pooled anxiety prevalence seen in survivors of neurocritical illness (37–43% depending on assessment tools used). In a meta-analysis evaluating 36 unique cohorts of critical illness survivors, post-traumatic stress symptoms were reported at 25–44% at 6 months and were still upward of 20% at 1 year [61]. Although our results here reveal a slightly lower disease burden of traumatic symptomatology, there were many fewer studies included in our analysis, all with follow-up ≤ 6 months. It is also important to note that many of the included studies rely on screening instruments rather than formal diagnosis. Therefore, the outcomes here reflect screening-positive symptoms rather than confirmed psychiatric illness.

It remains unclear how the macro-brain injury and micro-brain injury interact to produce the constellation of mental health outcomes seen in patients. A survivor with macro-injury to their nucleus accumbens might be expected to experience depressive symptoms [62]; another with injury to their locus coeruleus might experience decreased resilience [63]. However, patients who were in the general ICU without macro-brain injury also experience similar mental health outcomes [7]. Micro-injury mechanisms related to critical illness can be posited among patients with or without brain injury, including breakdown of the blood–brain barrier [64, 65], disrupted cerebral autoregulation [66], astrocyte activation [67], and changes in brain capillary density impacting nutrient delivery [68]. When an overall proinflammatory state in critical illness is combined with macro-injury-related structural lesions, there is an unsurprising impact on outcomes, including mental health. These mental health outcomes reciprocally interact with cognitive, quality-of-life, return-to-work, and physical outcomes [13]. It is crucial to understand that survivors with *good* motor outcomes can still face mental health challenges [69]. Therefore, a clinical imperative exists to examine these nonmotor outcomes that impact our patients’ lives.

While advances in intensive care medicine have improved survival, the persistent increased prevalence of anxiety, depression, and post-traumatic symptoms when compared with the general population highlights unmet needs for our survivors of neurocritical illness. In fact, Wintermann et al. highlighted the surprising number of patients who face undiagnosed mental health challenges [47]. Authors note the importance of continued improvements in accessible and personalized postdischarge rehabilitation care. Patients at high risk for mental health aspects of PICS are infrequently connected to appropriate follow-up care, resulting in barrier-prolonged psychological morbidity [70].

Interventions such as ICU diaries and ICU recovery clinics have been adopted to promote mental health following ICU discharge. ICU diaries are thought to impact mental health by introducing factual memories to replace aberrant or frightening memories [71]. However, these diaries have mixed impact on post-traumatic symptoms and depression, with some of the largest, randomized trials showing no benefit on psychological stress [61, 70, 72, 73]. The ways in which mental health interfaces with quality of life needs to be considered, in addition to the dyadic and reciprocal relationship that exists between patient and informal caregiver [4, 13, 32, 33, 43]. Moreover, the trajectory of survivors’ recovery needs to be considered, as early and late mental health symptoms may reflect acute versus chronic processes that pooled estimates could not distinguish [74, 75]. Future research studies should aim to include measures that are part of an established core outcomes set at standard timepoints to reduce heterogeneity in measurement tools and interpretation [76–80]. Prospective longitudinal cohort studies may also consider evaluating how underrepresented populations might be differentially impacted by neurocritical care illness. By assessing mental health outcomes immediately after discharge in our survivors of neurocritical illness, and monitoring changes in survivors longitudinally, we can have a clearer understanding of the efficacy of available rehabilitation interventions on both patient and caregiver [81].

This review has several strengths, including a systematic approach following established methods and reporting frameworks, and inclusion of geographically diverse studies, enhancing generalizability. Despite these strengths, our review had limitations. First, several risk-of-bias domains warrant consideration when interpreting these findings. Outcome assessment relied predominantly on self-report screening instruments rather than structured diagnostic interviews; therefore, our prevalence estimates reflect screening-positive symptoms rather than confirmed diagnoses. Second, loss to follow-up was the largest reason for risk of bias. Loss to follow-up in critically ill populations is often nonrandom, potentially introducing attrition and survivorship bias; for instance, patients with severe psychiatric symptoms may disengage, while those with severe neurological injuries may not survive to later timepoints, biasing estimates in either direction. Future studies should implement retention strategies and report attrition characteristics to enable bias-adjusted analyses. Further, the search strategy used in this systematic review may have introduced bias, as it focused on specific databases and previously used search terms [17]. Articles may have been missed during the search and screening steps; however, to minimize this risk, screening of abstracts was audited, with data abstraction performed in duplicate. The exclusion of non-English studies may also limit the generalizability. Studies in which neurological subgroup data could not be obtained were excluded, which may introduce selection bias if these studies systematically differed from those included. Another major limitation is the between-study heterogeneity; studies included a wide range of timepoints and variable tools, contributing to wide confidence intervals that limit interpretation. Other factors influencing heterogeneity include population-level factors, such as the age and sex of the populations included/excluded within each manuscript, diversity of diagnoses, and factors related to critical illness [82, 83]. Notably, prevalences following sensitivity analyses were similar, adding credence to the pooled prevalences seen when analyzing prevalences by other tools. Further, this limitation reflects the limitations of the literature itself, which precluded further subgroup analysis (e.g., by admission diagnosis category). Although the differing mechanisms of injury and clinical trajectories likely impact disease-specific mental health outcomes, this hypothesis was unable to be better elucidated by this analysis since pooling across diagnoses likely obscures disease-specific outcomes. Finally, while additional studies may have been published since the most recent search date, inclusion of these publications is unlikely to change the overall findings of this review.

## Conclusions

This review highlights the substantial burden of mental health symptoms in survivors of neurocritical illness. Despite significant between-study heterogeneity in measurement and follow-up, pooled estimates suggest that depression, anxiety, and post-traumatic stress are common, affecting—very approximately—one in four, one in three, and one in seven survivors after neurocritical care, respectively. These figures reflect approximate indicators of burden rather than precise prevalence estimates. Future studies should prioritize the use of standardized, validated instruments aligned with core outcome sets, as well as consistent follow-up timepoints, to reduce heterogeneity and improve comparability and synthesis of findings.

## Supplementary Material

sup

Supplementary Information

The online version contains supplementary material available at https://doi.org/10.1007/s12028-026-02576-2.

## Figures and Tables

**Table 1:** Summary of citations with mental health outcomes

First author and year (country)	Study design; population; max follow-up timepoint (months)^a,b^	Sample size (*n*), age (years), percentage (%) female^c^	Tools/methods for mental health outcomes assessment

Cuthbertson 2004 (Scotland)	Prospective; mixed; 3	n = 78; median age = 58; 44% F	DTS
Demir 2008 (Turkey)	Prospective; GBS; 6	*n* = 31; mean (SD) age = 54 (9); 39% F	New depression
Diaz 2012 (Brazil)	Prospective; TBI; 18	*n* = 33; mean (SD) age = 31 (11); 12% F	SCID-I, HADS, BPRS, AES
Forslund 2013 (Norway)	Prospective; TBI; 12	*n* = 85; mean (SD) age = 31 (11); 23% F	BDI
Geurts 2013 (Multiple)	RCT; CVA; 36	*n* = 33; 43% F	MADRS
Levine 2014 (USA)	Retrospective; CVA; 152.5	*n* = 370	CES-D
Ragoschke-Schumm 2015 (Multiple)	Retrospective; CVA; 93	*n* = 79; 39% F	HAM-D7, antidepressant use
Azouvi 2016 (France)	Prospective; TBI; 48	*n* = 85; mean (SD) age = 32 (13); 19% F	HADS
Khajavikhan 2016 (Iran)	Retrospective; TBI; 12	*n* = 40; mean(SD) age = 34 (17); 10% F	HADS
Bannon 2020 (USA)	RCT; CVA; 6	*n* = 16; mean (SD) age = 54 (17); 56% F	HADS, GAD-7, PCL-C, GSE
Liao 2020 (Taiwan)	Retrospective; mixed; 12	*n* = 90,655; mean age = 43; 41% F	New depression
Meyers 2020 (USA)^d^	Prospective; mixed; 6	*n* = 102; mean (SD) age = 52 (17); 47% F	HADS, PCL-5, MOCS-A
Bründl 2021 (Germany)	Prospective; SAH; 6	*n* = 21; mean age = 51; 62% F	ISR
Mikolić 2021 (Multiple)	Prospective; TBI; 6	*n* = 2064; mean age = 52; 33% F	PHQ-9, GAD-7, PCL-5
Presciutti 2021 (USA)	Prospective; mixed; 3	*n* = 70; mean age = 53; 46% F	PCL-5
Sadlonova 2021 (Germany)	RCT; CVA; 12	*n* = 360; mean (SD) age = 73 (8); 40% F	HADS
Zhao 2021 (China)	RCT; vascular malformation; 3	*n* = 54; median age = 26; 45% F	SAS, SDS
Bannon 2022 (USA)	Retrospective; mixed; 0, 1.5, 3	*n* = 58; mean age = 51; 52% F	HADS, PCL-C
Quinn 2023 (USA)	Prospective; mixed neuro; 0	*n* = 72; mean age = 52 (15); 55% F	HADS
Sonneville 2023 (France)	Prospective; CVA; 12	*n* = 174; median age = 62; 49.7%F	HADS
Wintermann 2023 (Germany)	Prospective; mixed; 6	*n* = 112; median age = 61; 53% F	ASDS, PTSS-10, SCID, MSPSS
Costello 2024 (Belgium)	Retrospective; NORSE; 6^e^	*n* = 39; median age = 30; 58% F	General mental health difficulties

AES, Apathy Evaluation Scale; ASDS, Acute Stress Disorder Scale; BDI, Beck Depression Inventory; BPRS, Brief Psychiatric Rating Scale; CES-D, Center for Epidemiologic Studies Depression Scale; CVA, cerebrovascular accident; DTS, Distress Tolerance Scale; F, female; GAD-7, General Anxiety Disorder-7; GSE, General Self-Efficacy Scale; GBS, Guillain-Barre syndrome; HADS, Hospital Anxiety and Depression Scale; HADS-A, Hospital Anxiety and Depression Scale, Anxiety Subscale; HADS-D, Hospital Anxiety and Depression Scale, Depression Subscale; HAM-D7, The Seven-Item Hamilton Depression Rating Scale; ISR, International Classification of Diseases Version 10 Symptom Rating; MADRS, Montgomery-Åsberg Depression Rating Scale; MOCS-A, Measure of Current Status, Part A; MSPSS, Multidimensional Scale of Perceived Social Support; NORSE, new onset refractory status epilepticus; PCL-5, Post-traumatic Stress Disorder Checklist for the Diagnostic and Statistical Manual of Mental Disorders, Fifth Edition; PCL-C, Post-traumatic Stress Disorder Checklist Civilian Version; PHQ-9, Patient Health Questionnaire-9; PTS, post-traumatic symptomatology; PTSS-10, Post-Traumatic Stress Symptom Scale-10; RCT, randomized controlled trial; SAH, subarachnoid hemorrhage; SAS, Zung Self-Rating Anxiety Scale; SDS, Zung Self-Rating Depression Scale; SCID-I, Structured Clinical Interview for Diagnostic and Statistical Manual of Mental Disorders, Fourth Edition, Axis I Disorders; SF-36-MH, Mental Health Subscale of Short-Form Survey-36; TBI, traumatic brain injury

a Follow-up duration converted to months and rounded to nearest whole number, using 30.44 as the average number of days in 1 month and 365.25 days in 1 year. Added if studies included this information

b “Mixed” refers to combined neurocritical care conditions, including cerebrovascular accident, trauma, spinal cord injury, or cancer. "Trauma" refers to trauma to body areas in addition to traumatic brain injury

c Sample size refers to participants available for mental health outcomes assessment, whereas age and percentage (%) female are provided for the entire study demographics, when available

d Three citations reporting on same population; all collapsed into single record here

e Results are reported for patients who survived at least 6 months, but range of survival 6–101 months following discharge

**Table 2:** Pooled prevalence (95%CI) of mental health outcomes at specific timepoints^a^

Post-ICU follow-up timepoint							Overall prevalence
	≤ 3 months	6 months	12 months	≥ 18 months	0–6 months	12 + months	

**Depression**							
*HADS-D only* ^b^							
No. studies	1	1	2	-	2	2	4
No. patients	72	102	162	-	174	162	336
Prevalence (95% CI)	25% (16–37%)	26% (18–36%)	23% (4–67%)	-	26% (18–36%)	23% (4–67%)	**24% (20–29%)**
I^2^	n/a	n/a	0%	-	0%	0%	0%
*All tools* ^c^							
No. studies	1	4	3	3	5	6	11
No. patients	72	2309	90,817	544	2381	91,361	93,742
Prevalence (95% CI)	25% (16–37%)	31% (10–64%)	16% (2–62%)	31% (3–85%)	30% (14–52%)	23% (10–45%)	**26% (16–38%)**
I^2^	n/a	92.8%	96.3%	90.8%	91.7%	98.1%	99.6%
*All validated* ^d^							
No. studies	1	3	2	2	4	4	8
No. patients	72	2278	162	465	2350	627	2977
Prevalence (95% CI)	25% (16–37%)	26% (4–72%)	23% (4–67%)	39% (0–100%)	26% (11–50%)	30% (11–60%)	**28% (18–41%)**
I^2^	n/a	95.0%	0%	95.1%	93.6%	86.0%	94.0%
*All validated tools without outlier studies* ^e^							
No. studies	1	3	2	1	4	3	7
No. patients	72	2278	162	432	2350	594	2944
Prevalence (95% CI)	25% (16–37%)	26% (4–72%)	23% (4–67%)	22% (18–26%)	26% (11–50%)	22% (18–27%)	**25% (17–34%)**
I^2^	n/a	95.0%	0%	n/a	93.6%	0%	94.5%
*All tools without Liao et al.* [39]^f^							
No. studies	1	4	2	3	5	5	10
No. patients	72	2309	162	544	2381	706	3087
Prevalence (95% CI)	25% (16–37%)	31% (10–64%)	23% (4–67%)	31% (3–85%)	30% (14–52%)	28% (12–49%)	**29% (20–40%)**
I^2^	n/a	92.8%	0%	90.8%	91.7%	82.2%	93.1%
*All tools without outlier studies^e^*							
No. studies	1	3	2	2	4	4	8
No. patients	72	245	162	511	317	673	990
Prevalence (95% CI)	25% (16–37%)	27% (3–81%)	23% (4–67%)	22% (11–38%)	26% (9–56%)	22% (19–25%)	**24% (17–32%)**
I^2^	n/a	89.7%	0%	0%	84.5%	0%	67.7%
**Anxiety**							
*HADS-A only^b^*							
No. studies	2	2	2	-	4	2	6
No. patients	428	116	214	-	544	214	758
Prevalence (95% CI)	25% (0–100%)	43% (0–99%)	45% (5–93%)	-	33% (11–68%)	45% (5–93%)	**37% (21–56%)**
I^2^	96.6%	53.9%	40.0%	-	93.8%	40.0%	93.9%
*All toolsc*							
No. studies	2	3	2	-	5	2	7
No. patients	428	2175	214	-	2603	214	2817
Prevalence (95% CI)	25% (0–100%)	32% (4–85%)	45% (5–93%)	-	28% (11–54%)	45% (5–93%)	**32% (18–51%)**
I^2^	96.6%	95.4%	40.0%	-	94.6%	40.0%	96.6%
*All tools without outlier studiese*							
No. studies	1	2	2	-	3	2	5
No. patients	72	116	214	-	188	214	402
Prevalence (95% CI)	42% (30–54%)	43% (0–99%)	45% (5–93%)	-	40% (24–58%)	45% (5–93%)	**43% (35–52%)**
I^2^	n/a	53.9%	40.0%	-	13.7%	40.0%	32.0%
**Post-traumatic stress** ^g^							
*All tools*							
No. studies	4	2	-	-	6	-	6
No. patients	420	2153	-	-	2573	-	2573
Prevalence (95% CI)	14% (9–20%)	15% (0–99%)	-	-	14% (8–21%)	-	**14% (8–21%)**
I^2^	0%	94.3%	-	-	79.0%	-	79.0%

Bold values indicate the prevalence for that specific subset

BDI, Beck Depression Inventory; CES-D, Center for Epidemiologic Studies Depression Scale; CI, confidence interval; HADS-A, Hospital Anxiety and Depression Scale, Anxiety Subscale; HADS-D, Hospital Anxiety and Depression Scale, Depression Subscale; HAM-D, Hamilton Depression Rating Scale; MADRS, Montgomery-Åsberg Depression Rating Scale; No., number; PHQ-9, Patient Health Questionnaire-9

a “General mental health” outcome not included due to lack of data that could be pooled

b Significant disease defined in all studies as HADS-D and HADS-A of ≥ 7 or ≥ 8. Also see Supplementary Figs. S1 (Depression) and S5 (Anxiety)

c All assessments, including nonvalidated depression measures such as "Depression tendency" and "new antidepressant use." All anxiety assessments performed were validated. See Supplementary Figs. S2 (Depression) and S6 (Anxiety)

d Validated depression scales included HADS-D, PHQ-9, BDI, HAM-D, structured interview, CES-D, and MADRS. Also see Supplementary Table S3 and Supplementary Fig. S3

e “Outlier” defined as a study having its individual 95% CI outside the overall prevalence 95% CI. Also see Supplementary Figs. S2e–f and S3c–d (Depression) and S6c–d (Anxiety)

f Liao et al. study excluded due to substantial sample size (n = 90.655) and nonvalidated depression tool. Also see Supplementary Fig. S2c–d

g PCL-C subgroup (*n* = 2 studies) excluded from Table 2 due to insufficient data for pooling. Mean PCL-C score of 36.3 (95% CI 13.0–59.6; *I*^2^ = 20%) is consistent with moderate to moderately high post-traumatic stress symptoms. Also see Supplementary Fig. S8
