# Supplementary material for: Mental Health Outcomes After Neurocritical Care: A Systematic Review and Meta-analysis
Source: Neurocrit Care. Author manuscript; Available in PMC 2026 Aug 20. (PMC13492101; doi:10.1007/s12028-026-02576-2)
Supplement: Supplementary file 1 [file NIHMS2196051-supplement-sup.pdf]

SUPPLEMENTAL DIGITAL CONTENT

Mental Health Outcomes after Neurocritical Care: A Systematic Review and Meta-Analysis

Jamie Nicole LaBuzetta MD, MSc, MPhil[1]; Henry G. Chen, BA[2]; Nicholas Ibrahim, BS[1]; Onyekachi Ezeokeke, BS[2]; Sanil Gandhi, MD[3]; Victor D. Dinglas, MPH[4,5]; Dale M. Needham, MD, PhD[4,5,6]; Biren B. Kamdar MD, MBA, MHS[7,8]

Affiliations

1 Department of Neurosciences, Division of Neurocritical Care, UC San Diego Health, University of California, San Diego, La Jolla, CA, USA
2 School of Medicine, University of California, San Diego, La Jolla, CA, USA
3 Department of Medicine, UC Irvine School of Medicine, University of California, Irvine, CA, USA
4 Division of Pulmonary and Critical Care Medicine, School of Medicine, Johns Hopkins University, Baltimore, Maryland, USA
5 Outcomes After Critical Illness and Surgery (OACIS) Research Group, Johns Hopkins University, Baltimore, Maryland, USA
6 Department of Physical Medicine and Rehabilitation, School of Medicine, Johns Hopkins University, Baltimore, Maryland, USA
7 Department of Medicine, Division of Pulmonary, Critical Care and Sleep Medicine, UC San Diego Health, University of California, San Diego, La Jolla, CA, USA
8 Department of Medicine, VA San Diego Healthcare System, La Jolla, CA, USA

Table of Contents

1. **Supplemental Table S1.** PRISMA Checklist
2. **Supplemental Table S2.** Search terms utilized across multiple searches for identification of eligible studies
3. **Supplemental Table S3.** Characteristics of manuscripts reporting on depression outcomes
4. **Supplemental Figure S1.** Forest Plot: Depression Prevalence in Studies Using HADS-D
5. **Supplemental Figure S2.** Forest Plot: Depression Prevalence in All Studies
6. **Supplemental Figure S3.** Forest Plot: Depression Prevalence in Studies Using Validated Tools to Measure Depression
7. **Supplemental Figure S4.** Forest Plot: Pooled HADS-D Scores for Depression Symptoms
8. **Supplemental Table S4.** Characteristics of manuscripts reporting on anxiety outcomes
9. **Supplemental Figure S5.** Forest Plot: Anxiety Prevalence in Studies Using HADS-A
10. **Supplemental Figure S6.** Forest Plot: Anxiety Prevalence in All Studies
11. **Supplemental Figure S7.** Forest Plot: Pooled HADS-A Scores for Anxiety Symptoms
12. **Supplemental Table S5.** Characteristics of manuscripts reporting on post-traumatic outcomes
13. **Supplemental Figure S8.** Forest Plot: Post-Traumatic Stress Prevalence
14. **Supplemental Figure S9.** Forest Plot: Pooled PCL-C Scores for Post-Traumatic Stress Symptoms
15. **Supplemental Table S6.** Characteristics of manuscripts reporting on general mental health outcomes
16. **Supplemental Table S7.** Risk of Bias

Supplemental Table S1. PRISMA Checklist

Section and Topic	Item #	Checklist item	Location in Manuscript
**TITLE**			
Title	1	Identify the report as a systematic review.	Page 1
**ABSTRACT**			
Abstract	2	See the PRISMA 2020 for Abstracts checklist.	Page 3
**INTRODUCTION**			
Rationale	3	Describe the rationale for the review in the context of existing knowledge.	Page 4
Objectives	4	Provide an explicit statement of the objective(s) or question(s) the review addresses.	Page 4
**METHODS**			
Eligibility criteria	5	Specify the inclusion and exclusion criteria for the review and how studies were grouped for the syntheses.	Page 6. "Study Selection" Section
Information sources	6	Specify all databases, registers, websites, organisations, reference lists and other sources searched or consulted to identify studies. Specify the date when each source was last searched or consulted.	Page 5. "Identification of Eligible Studies" Section
Search strategy	7	Present the full search strategies for all databases, registers and websites, including any filters and limits used.	Supplemental Table S2
Selection process	8	Specify the methods used to decide whether a study met the inclusion criteria of the review, including how many reviewers screened each record and each report retrieved, whether they worked independently, and if applicable, details of automation tools used in the process.	Page 6. "Study Selection" Section
Data collection process	9	Specify the methods used to collect data from reports, including how many reviewers collected data from each report, whether they worked independently, any processes for obtaining or confirming data from study investigators, and if applicable, details of automation tools used in the process.	Pages 6-7. "Data Abstraction and Summarization" Section
Data items	10a	List and define all outcomes for which data were sought. Specify whether all results that were compatible with each outcome domain in each study were sought (e.g. for all measures, time points, analyses), and if not, the methods used to decide which results to collect.	Page 7. "Data Abstraction and Summarization" Section
	10b	List and define all other variables for which data were sought (e.g. participant and intervention characteristics, funding sources). Describe any assumptions made about any missing or unclear information.	Page 7. "Data Abstraction and Summarization" Section
Study risk of bias assessment	11	Specify the methods used to assess risk of bias in the included studies, including details of the tool(s) used, how many reviewers assessed each study and whether they worked independently, and if applicable, details of automation tools used in the process.	Page 6
Effect measures	12	Specify for each outcome the effect measure(s) (e.g. risk ratio, mean difference) used in the synthesis or presentation of results.	Page 7
Synthesis methods	13a	Describe the processes used to decide which studies were eligible for each synthesis (e.g. tabulating the study intervention characteristics and comparing against the planned groups for each synthesis (item #5)).	Pages 5-6. "Study Selection"
	13b	Describe any methods required to prepare the data for presentation or synthesis, such as handling of missing summary statistics, or data conversions.	Pages 6-7. "Data Abstraction and Summarization" Section
	13c	Describe any methods used to tabulate or visually display results of individual studies and syntheses.	Pages 6-8. "Data Abstraction and Summarization" and "Meta-analysis" sections
	13d	Describe any methods used to synthesize results and provide a rationale for the choice(s). If meta-analysis was performed, describe the model(s), method(s) to identify the presence and extent of statistical heterogeneity, and software package(s) used.	Pages 7-9. "Meta-analysis" section
	13e	Describe any methods used to explore possible causes of heterogeneity among study results (e.g. subgroup analysis, meta-regression).	Pages 8-9. "Meta-analysis" section

Section and Topic	Item #	Checklist item	Location in Manuscript
	13f	Describe any sensitivity analyses conducted to assess robustness of the synthesized results.	Page 8. "Meta-analysis" section
Reporting bias assessment	14	Describe any methods used to assess risk of bias due to missing results in a synthesis (arising from reporting biases).	Page 6
Certainty assessment	15	Describe any methods used to assess certainty (or confidence) in the body of evidence for an outcome.	Page 8. "Meta-analysis" section
**RESULTS**			
Study selection	16a	Describe the results of the search and selection process, from the number of records identified in the search to the number of studies included in the review, ideally using a flow diagram.	Page 9. Figure 1
	16b	Cite studies that might appear to meet the inclusion criteria, but which were excluded, and explain why they were excluded.	Page 9. Figure 1
Study characteristics	17	Cite each included study and present its characteristics.	Table 1
Risk of bias in studies	18	Present assessments of risk of bias for each included study.	Supp Table S7
Results of individual studies	19	For all outcomes, present, for each study: (a) summary statistics for each group (where appropriate) and (b) an effect estimate and its precision (e.g. confidence/credible interval), ideally using structured tables or plots.	Table 2; Tables S3, S4, S5, S6
Results of syntheses	20a	For each synthesis, briefly summarise the characteristics and risk of bias among contributing studies.	Table S7
	20b	Present results of all statistical syntheses conducted. If meta-analysis was done, present for each the summary estimate and its precision (e.g. confidence/credible interval) and measures of statistical heterogeneity. If comparing groups, describe the direction of the effect.	Pages 10-12<br>Figures S1-S9
	20c	Present results of all investigations of possible causes of heterogeneity among study results.	Figures S1-S9
	20d	Present results of all sensitivity analyses conducted to assess the robustness of the synthesized results.	Page 10-11
Reporting biases	21	Present assessments of risk of bias due to missing results (arising from reporting biases) for each synthesis assessed.	Not Presented
Certainty of evidence	22	Present assessments of certainty (or confidence) in the body of evidence for each outcome assessed.	Table 2<br>Figures S1-S9
**DISCUSSION**			
Discussion	23a	Provide a general interpretation of the results in the context of other evidence.	Pages 13-15
	23b	Discuss any limitations of the evidence included in the review.	Page 17
	23c	Discuss any limitations of the review processes used.	Page 17
	23d	Discuss implications of the results for practice, policy, and future research.	Pages 15-16
**OTHER INFORMATION**			
Registration and protocol	24a	Provide registration information for the review, including register name and registration number, or state that the review was not registered.	Page 5
	24b	Indicate where the review protocol can be accessed, or state that a protocol was not prepared.	Page 5
	24c	Describe and explain any amendments to information provided at registration or in the protocol.	N/A
Support	25	Describe sources of financial or non-financial support for the review, and the role of the funders or sponsors in the review.	Page 2
Competing interests	26	Declare any competing interests of review authors.	Page 2
Availability of data, code and other materials	27	Report which of the following are publicly available and where they can be found: template data collection forms; data extracted from included studies; data used for all analyses; analytic code; any other materials used in the review.	Not Reported

Supplemental Table S2: Search terms utilized across multiple searches for identification of eligible studies	
**Database**	**Search Terms**
Pubmed	(intensive care[tiab] OR intensive therapy[tiab] OR high dependency[tiab] OR critical care[tiab] OR "critical care"[MeSH Terms] OR intermediate care[tiab] OR step-up care[tiab] OR step-down care[tiab] OR respiratory distress syndrome[tiab] OR acute lung injury[tiab]) AND (outcome measure[tiab] OR "outcome assessment ,health care"[MeSH Terms] OR follow-up[tiab] OR "follow-up studies"[MeSH Terms] OR health status[tiab] OR "health status"[MeSH Terms] OR functional status[tiab] OR clinical outcome[tiab]) AND (organ failure[tiab] OR "multiple organ failure"[MeSH Terms] OR organ dysfunction[tiab] OR sequelae[tiab] OR quality of life[tiab] OR "quality of life"[MeSH Terms] OR impairment[tiab] OR morbidity[tiab] OR "morbidity"[MeSH Terms]) NOT (animals[mh] NOT humans[mh])<br>**Limits:** 1970 to 06-30-2024
Embase	('intensive care'/exp OR 'intensive care':ti,ab OR 'intensive therapy':ti,ab OR 'high dependency':ti,ab OR 'critical care':ti,ab OR 'critical care':ti,ab OR 'intermediate care':ti,ab OR 'step-up care':ti,ab OR 'step-down care':ti,ab OR 'respiratory distress syndrome':ti,ab OR 'acute lung injury':ti,ab) AND ('outcome measure':ti,ab OR 'outcome assessment'/exp OR 'follow up':ti,ab OR 'follow up'/exp OR 'health status':ti,ab OR 'health status'/de OR 'functional status':ti,ab OR 'clinical outcome':ti,ab) AND ('organ failure':ti,ab OR 'multiple organ failure'/exp OR 'organ dysfunction':ti,ab OR 'multiple organ failure':ti,ab OR 'sequelae':ti,ab OR 'quality of life'/exp OR 'quality of life':ti,ab OR 'impairment':ti,ab OR 'morbidity':ti,ab OR 'morbidity'/exp) NOT ('animal'/exp NOT ('animal'/exp AND 'human'/exp))<br>**Limits:** 1970 to 06-30-2024
CINAHL	(MH "intensive care units+" OR "intensive care" OR "intensive therapy" OR "high dependency" OR MH "critical care" OR "critical care" OR "intermediate care" OR "step-up care" OR "step-down care" OR "respiratory distress syndrome" OR "acute lung injury") AND ("outcome measure" OR MH "outcome assessment" OR "follow up" OR MH "prospective studies" OR MH "health status" OR "health status" OR "functional status" OR "clinical outcome") AND ("organ failure" OR "multiple organ failure" OR "organ dysfunction" OR MH "multiple organ dysfunction syndrome" OR "sequelae" OR "quality of life" OR MH "quality of life" OR "impairment" OR "morbidity" OR MH "morbidity")<br>**Limits:** 1970 to 06-30-2024
PsycInfo	(DE "intensive care" OR "intensive care" OR "intensive therapy" OR "high dependency" OR "critical care" OR "intermediate care" OR "step-up care" OR "step-down care" OR "respiratory distress syndrome" OR "acute lung injury") AND ("outcome measure" OR "outcome assessment" OR "follow up" OR DE "Followup Studies" OR "health status" OR "functional status" OR "clinical outcome") AND ("organ failure" OR "multiple organ failure" OR "organ dysfunction" OR "sequelae" OR "quality of life" OR DE "quality of life" OR "impairment" OR DE "morbidity" OR "morbidity")<br>**Limits:** 1970 to 06-30-2024

Supplemental Table S3: Characteristics of manuscripts reporting on depression outcomes

Citation	Instrument Used	Study *N* at Follow-Up	Follow-Up Timeline (Mo)	Depression Prevalence (%)	Depression Score Mean (SD)
Meyers 2020 [a]	HADS-D	102	0<br>3<br>6	23<br>24<br>26	5.0 (4.7)<br>4.6 (4.9)<br>4.8 (5.4)
Bannon 2022	HADS-D	58<br>52<br>49	0<br>1.75<br>3	NR	7.9 (4.1)<br>5.6 (5.6)<br>5.7 (4.2)
Sadlonova 2021	HADS-D	360	0<br>3<br>12	NR	4.4 (4.4)<br>(--) [h]<br>(--) [h]
Bannon 2020	HADS-D	16	0<br>1.5<br>3	NR	6.3 (4.2)<br>10.1 (3.5)<br>8.6 (2.9)
Azouvi 2016	HADS-D	118	48	NR	5.2 (4.5) i
Khajavikhan 2016	HADS-D	40	12	27.5	NR
Quinn 2023	HADS-D	72	0	25	4.5 (4.1)
Sonneville 2023	HADS-D	122	12	21.3	7 (4, 11) [j]
Mikolić 2021	PHQ-9	2064	6	42	NR
Diaz 2012	AES	33	18	NR	28.4 (14.9)
Wintermann 2023	SCID-1	112	3<br>6	8.3<br>12.2	NR
Levine 2014	CES-D	370 [k]	152.5 [d]	22	NR
Forslund 2013	BDI	85	12	NR	10.7 (8.6)
Geurts 2013	MADRS [b]	33	36	61 [e]	NR
Demir 2008	Depression Tendency	31	6	52 [f]	NR
Liao 2020	New depression	90,655	12	7	NR
Ragoschke-Schumm 2015	HAMD-7<br>-----------<br>Antidepressant Use	79	93 [d]	(--)<br>-----------<br>19 [g]	4.2 (2.7)<br>-----------<br>(--)
Zhao 2021	SDS [b]	54 [c]	3	NR	1.1 (0.88)

Abbreviations: AES: Apathy Evaluation Scale; BDI: Beck Depression inventory; CES-D: Center for Epidemiologic Studies Depression Scale; HADS-D: Hospital Anxiety and Depression Scale, Depression Subscale; HAMD-7: The 7-Item Hamilton Depression Rating Scale; MADRS: Montgomery-Åsberg Depression Rating Scale; NR: not reported; PHQ-9: Patient Health Questionnaire-9; SCID-1: Structured interview with diagnosis of major depression; SDS: Zung Self-Rating Depression Scale

[a] Multiple citations reporting on same population collapsed into single record here.
[b] Measures severity of depression.
[c] n=54 in control group, and data specific to non-intervention group.
[d] Retrospective with variable follow-up; max follow-up included here.
[e] Prevalence of moderate-to-severe depression.
[f] n=16 participants described as having a "tendency towards depression" by a psychiatrist.
[g] Recorded as a percent of participants prescribed antidepressants, n=15.
[h] Data presented graphically for t=3mo and t=12mo with value between 4.25 and 4.00; exact values not available.
[i] Based upon personal communication with the author.
[j] Data presented as median (IQR).
[k] n=370 participants representing 432 stroke events/hospitalizations.

Supplemental Figure S1:

Figure S1a:

Forest Plot: Depression Prevalence in Studies Using HADS-D at ≤3, 6, and ≥12 months

Study or Subgroup	Events	Total	Weight	IV, Random, 95% CI
**Subgroup = 0-3 months**				
Quinn 2023	18	72	21.8%	0.25 [0.16; 0.37]
**Subgroup = 6 months**				
Meyers 2020	27	102	32.1%	0.26 [0.18; 0.36]
**Subgroup = 12 months**				
Khajavikhan 2016	11	40	12.9%	0.28 [0.15; 0.44]
Sonneville 2023	26	122	33.1%	0.21 [0.14; 0.30]
**Total (95% CI)**		**162**	**46.0%**	**0.23 [0.04; 0.67]**
Heterogeneity: Tau^2 = 0; Chi^2 = 0.65, df = 1 (P = 0.4197); I^2 = 0%				
**Total (95% CI)**		**336**	**100.0%**	**0.24 [0.20; 0.29]**
**Prediction interval**				**[0.18; 0.33]**

IV, Random, 95% CI

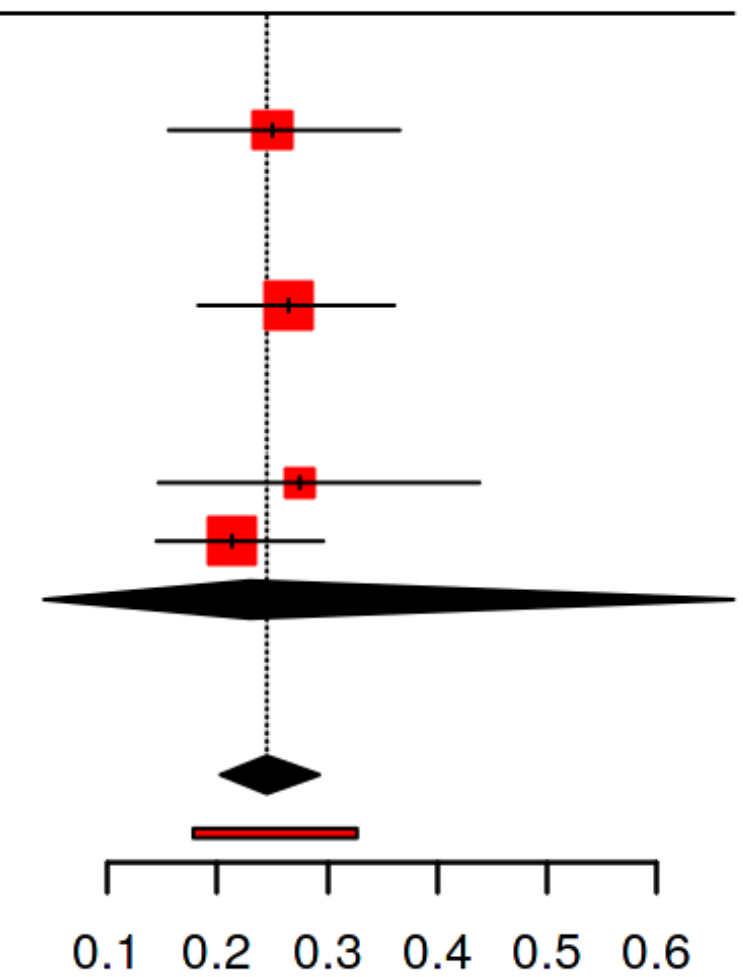

Heterogeneity: Tau^2 = 0; Chi^2 = 1.09, df = 3 (P = 0.7803); I^2 = 0.0%
Test for subgroup differences: Chi^2 = 0.53, df = 2 (P = 0.7689)

Footnotes:
[1] Four studies analyzed totaling 336 subjects.
[2] HADS-D cutoff ≥8 for all studies except Quinn 2023, which used ≥7.
[3] Based on the analysis performed using a random effects model with the inverse variance method and logit transformation, the summarized proportion is 0.24 with a 95% confidence interval of 0.20-0.29.
[4] Notable variability not detected, signaling that the effect sizes across cohorts were uniform in both size and direction.

Figure S1b:

Forest Plot: Depression Prevalence in Studies Using HADS-D at ≤6 and ≥12 months

Study or Subgroup	Events	Total	Weight	IV, Random, 95% CI
**Subgroup = 0-6 months**				
Quinn 2023	18	72	21.8%	0.25 [0.16; 0.37]
Meyers 2020	27	102	32.1%	0.26 [0.18; 0.36]
**Total (95% CI)**		**174**	**54.0%**	**0.26 [0.18; 0.36]**
Heterogeneity: $Tau^2 = 0$; $Chi^2 = 0.05$, df = 1 (P = 0.8273); $I^2 = 0\%$				
**Subgroup = 12 months**				
Khajavikhan 2016	11	40	12.9%	0.28 [0.15; 0.44]
Sonneville 2023	26	122	33.1%	0.21 [0.14; 0.30]
**Total (95% CI)**		**162**	**46.0%**	**0.23 [0.04; 0.67]**
Heterogeneity: $Tau^2 = 0$; $Chi^2 = 0.65$, df = 1 (P = 0.4197); $I^2 = 0\%$				
**Total (95% CI)**		**336**	**100.0%**	**0.24 [0.20; 0.29]**
**Prediction interval**				**[0.18; 0.33]**

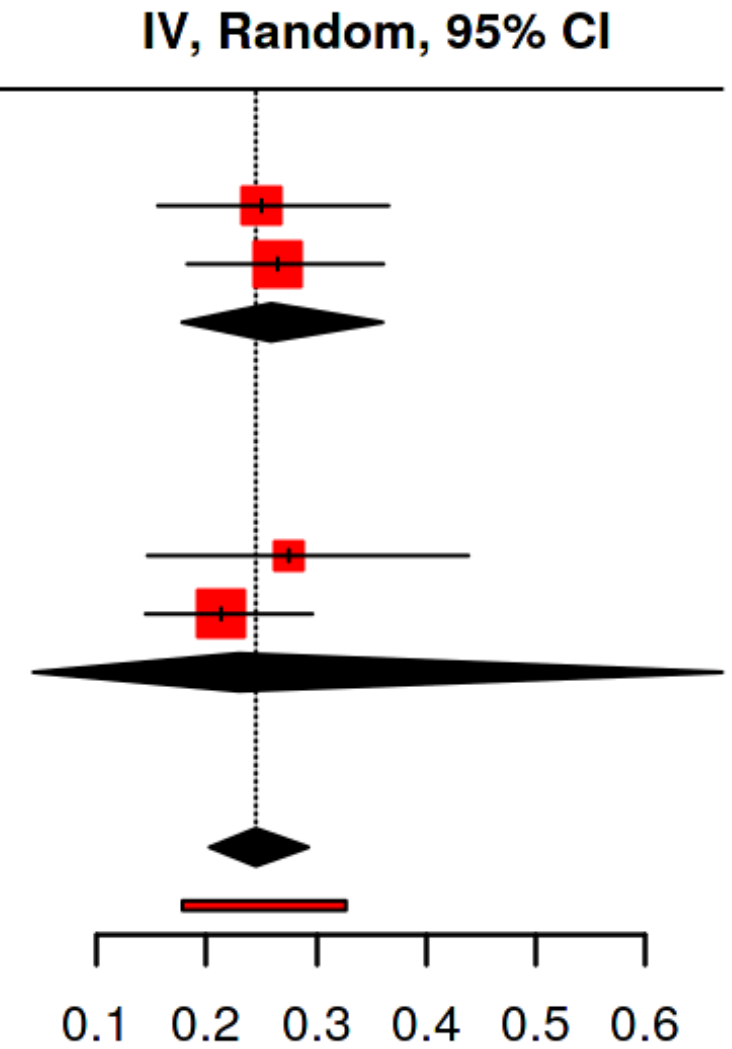

Heterogeneity: $Tau^2 = 0$; $Chi^2 = 1.09$, df = 3 (P = 0.7803); $I^2 = 0.0\%$
Test for subgroup differences: $Chi^2 = 1.04$, df = 1 (P = 0.3081)

Footnotes:

[1] Four studies analyzed totaling 336 subjects.

[2] HADS-D cutoff ≥8 for all studies except Quinn 2023, which used ≥7.

[3] Based on the analysis performed using a random effects model with the inverse variance method and logit transformation, the summarized proportion is 0.24 with a 95% confidence interval of 0.20-0.29.

[4] Notable variability not detected, signaling that the effect sizes across cohorts were uniform in both size and direction.

Supplemental Figure S2:

Figure S2a:

Forest Plot: Depression Prevalence in All Studies at ≤3, 6, 12, ≥18months

Study or Subgroup	Events	Total	Weight	IV, Random, 95% CI
**Subgroup = 0-3 months**				
Quinn 2023	18	72	9.1%	0.25 [0.16; 0.37]
**Subgroup = 6 months**				
Demir 2008	16	31	8.8%	0.52 [0.33; 0.70]
Meyers 2020	27	102	9.2%	0.26 [0.18; 0.36]
Mikolić 2021	867	2064	9.4%	0.42 [0.40; 0.44]
Wintermann 2023	14	112	9.0%	0.12 [0.07; 0.20]
**Total (95% CI)**		**2309**	**36.4%**	**0.31 [0.10; 0.64]**
Heterogeneity: $Tau^2 = 0.5076$; $Chi^2 = 41.4$, df = 3 ($P < 0.0001$); $I^2 = 92.8\%$				
**Subgroup = 12 months**				
Khajavikhan 2016	11	40	8.9%	0.28 [0.15; 0.44]
Liao 2020	6346	90655	9.4%	0.07 [0.07; 0.07]
Sonneville 2023	26	122	9.2%	0.21 [0.14; 0.30]
**Total (95% CI)**		**90817**	**27.4%**	**0.16 [0.02; 0.62]**
Heterogeneity: $Tau^2 = 0.9204$; $Chi^2 = 54.14$, df = 2 ($P < 0.0001$); $I^2 = 96.3\%$				
**Subgroup = 18+ months**				
Geurts 2013	20	33	8.8%	0.61 [0.42; 0.77]
Ragoschke-Schumm 2015	15	79	9.0%	0.19 [0.11; 0.29]
Levine 2014	95	432	9.3%	0.22 [0.18; 0.26]
**Total (95% CI)**		**544**	**27.2%**	**0.31 [0.03; 0.85]**
Heterogeneity: $Tau^2 = 0.5894$; $Chi^2 = 21.79$, df = 2 ($P < 0.0001$); $I^2 = 90.8\%$				
**Total (95% CI)**		**93742**	**100.0%**	**0.26 [0.16; 0.38]**
**Prediction interval**				**[0.01; 0.91]**

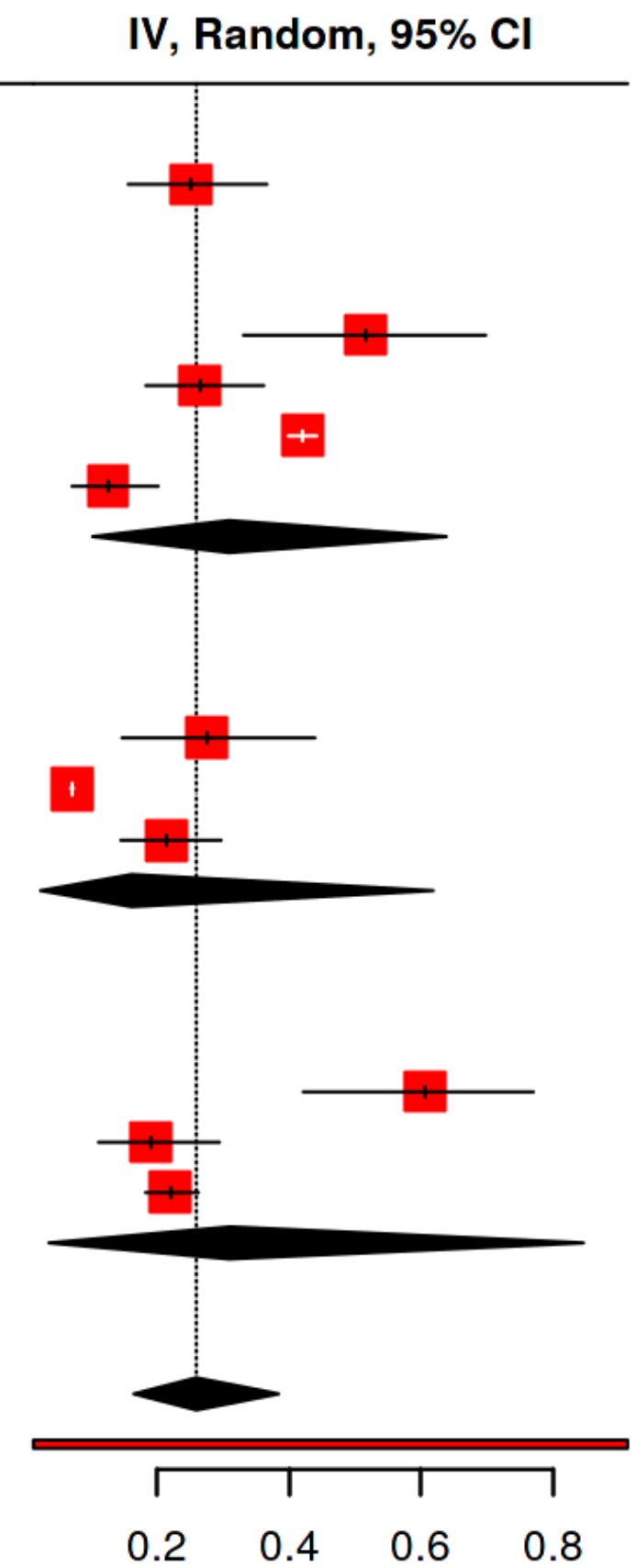

Heterogeneity: $Tau^2 = 2.1490$; $Chi^2 = 2691.88$, df = 10 ($P = 0$); $I^2 = 99.6\%$
Test for subgroup differences: $Chi^2 = 1.96$, df = 3 ($P = 0.5817$)

Footnotes:
[1] Eleven studies analyzed totaling 93,742 subjects.
[2] Only last timepoint data was included from studies with multiple timepoints of follow-up. Data from all timepoints can be found in Table S3.
[3] Based on the analysis performed using a random effects model with the inverse variance method and logit transformation, the summarized proportion is 0.26 with a 95% confidence interval of 0.16-0.38.
[4] Significant heterogeneity detected (p<0.01), suggesting inconsistent effects in magnitude and/or direction. I^2 value indicates 100% of variability among studies arising from heterogeneity rather than random chance.

Figure S2b:

Forest Plot: Depression Prevalence in All Studies at ≤6 and ≥12months

Study or Subgroup	Events	Total	Weight	IV, Random, 95% CI
**Subgroup = 0-6 months**				
Quinn 2023	18	72	9.1%	0.25 [0.16; 0.37]
Demir 2008	16	31	8.8%	0.52 [0.33; 0.70]
Meyers 2020	27	102	9.2%	0.26 [0.18; 0.36]
Mikolić 2021	867	2064	9.4%	0.42 [0.40; 0.44]
Wintermann 2023	14	112	9.0%	0.12 [0.07; 0.20]
**Total (95% CI)**		**2381**	**45.4%**	**0.30 [0.14; 0.52]**
Heterogeneity: $Tau^2 = 0.4415$; $Chi^2 = 48.22$, df = 4 ($P < 0.0001$); $I^2 = 91.7\%$				
**Subgroup = 12+ months**				
Khajavikhan 2016	11	40	8.9%	0.28 [0.15; 0.44]
Liao 2020	6346	90655	9.4%	0.07 [0.07; 0.07]
Sonneville 2023	26	122	9.2%	0.21 [0.14; 0.30]
Geurts 2013	20	33	8.8%	0.61 [0.42; 0.77]
Ragoschke-Schumm 2015	15	79	9.0%	0.19 [0.11; 0.29]
Levine 2014	95	432	9.3%	0.22 [0.18; 0.26]
**Total (95% CI)**		**91361**	**54.6%**	**0.23 [0.10; 0.45]**
Heterogeneity: $Tau^2 = 1.0801$; $Chi^2 = 265.97$, df = 5 ($P < 0.0001$); $I^2 = 98.1\%$				
**Total (95% CI)**		**93742**	**100.0%**	**0.26 [0.16; 0.38]**
**Prediction interval**				**[0.01; 0.91]**

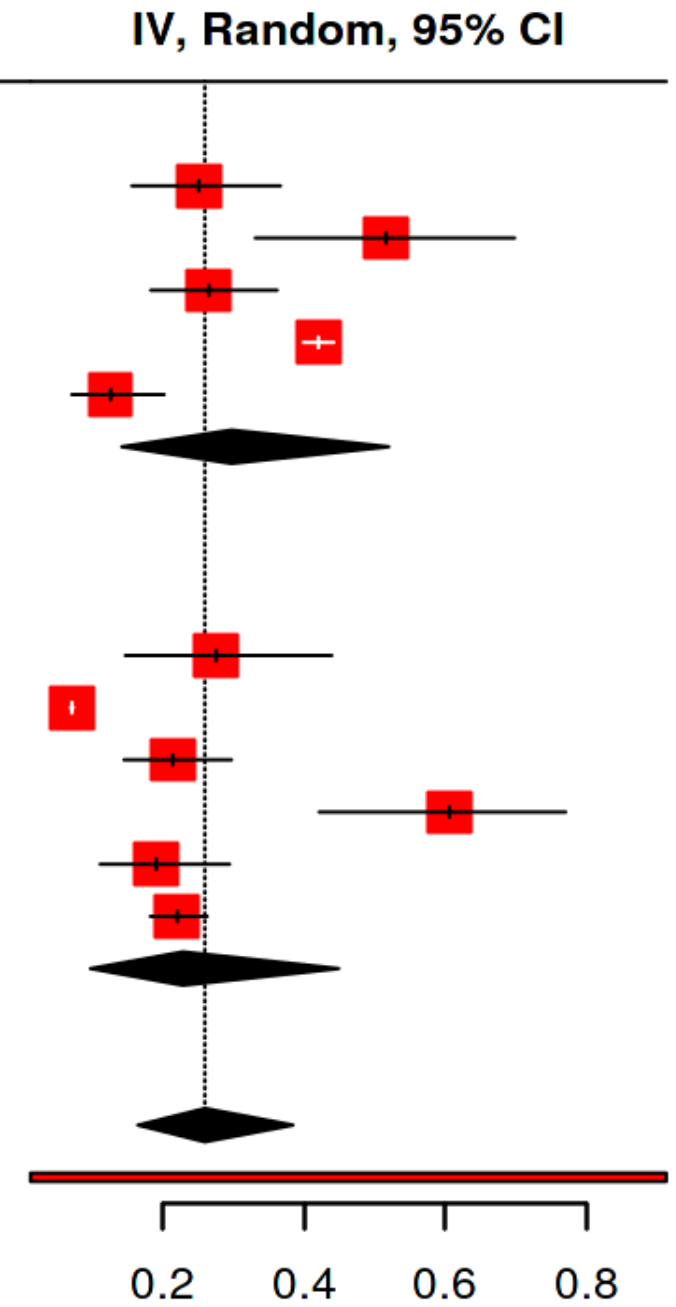

Heterogeneity: $Tau^2 = 2.1490$; $Chi^2 = 2691.88$, df = 10 ($P = 0$); $I^2 = 99.6\%$
Test for subgroup differences: $Chi^2 = 0.47$, df = 1 ($P = 0.4924$)

Footnotes:

[1] Eleven studies analyzed totaling 93,742 subjects.

[2] Only last timepoint data was included from studies with multiple timepoints of follow-up. Data from all timepoints can be found in Table E3.

[3] Based on the analysis performed using a random effects model with the inverse variance method and logit transformation, the summarized proportion is 0.26 with a 95% confidence interval of 0.16-0.38.

[4] Significant heterogeneity detected (p<0.01), suggesting inconsistent effects in magnitude and/or direction. The I^2 value indicates that 100% of the variability among studies arises from heterogeneity rather than random chance.

Figure S2c:

Forest Plot: Depression Prevalence in All Studies at ≤3, 6, 12, ≥18months *without Liao et al.*

Study or Subgroup	Events	Total	Weight	IV, Random, 95% CI
**Subgroup = 0-3 months**				
Quinn 2023	18	72	9.9%	0.25 [0.16; 0.37]
**Subgroup = 6 months**				
Demir 2008	16	31	8.9%	0.52 [0.33; 0.70]
Meyers 2020	27	102	10.4%	0.26 [0.18; 0.36]
Mikolić 2021	867	2064	11.6%	0.42 [0.40; 0.44]
Wintermann 2023	14	112	9.8%	0.12 [0.07; 0.20]
**Total (95% CI)**		**2309**	**40.7%**	**0.31 [0.10; 0.64]**
Heterogeneity: $Tau^2 = 0.5076$; $Chi^2 = 41.4$, df = 3 ($P < 0.0001$); $I^2 = 92.8\%$				
**Subgroup = 12 months**				
Khajavikhan 2016	11	40	9.0%	0.28 [0.15; 0.44]
Sonneville 2023	26	122	10.5%	0.21 [0.14; 0.30]
**Total (95% CI)**		**162**	**19.4%**	**0.23 [0.04; 0.67]**
Heterogeneity: $Tau^2 = 0$; $Chi^2 = 0.65$, df = 1 ($P = 0.4197$); $I^2 = 0\%$				
**Subgroup = 18+ months**				
Geurts 2013	20	33	8.9%	0.61 [0.42; 0.77]
Ragoschke-Schumm 2015	15	79	9.7%	0.19 [0.11; 0.29]
Levine 2014	95	432	11.3%	0.22 [0.18; 0.26]
**Total (95% CI)**		**544**	**30.0%**	**0.31 [0.03; 0.85]**
Heterogeneity: $Tau^2 = 0.5894$; $Chi^2 = 21.79$, df = 2 ($P < 0.0001$); $I^2 = 90.8\%$				
**Total (95% CI)**		**3087**	**100.0%**	**0.29 [0.20; 0.40]**
**Prediction interval**				**[0.08; 0.65]**

Heterogeneity: $Tau^2 = 0.4078$; $Chi^2 = 129.70$, df = 9 ($P < 0.0001$); $I^2 = 93.1\%$

Test for subgroup differences: $Chi^2 = 1.17$, df = 3 ($P = 0.7603$)

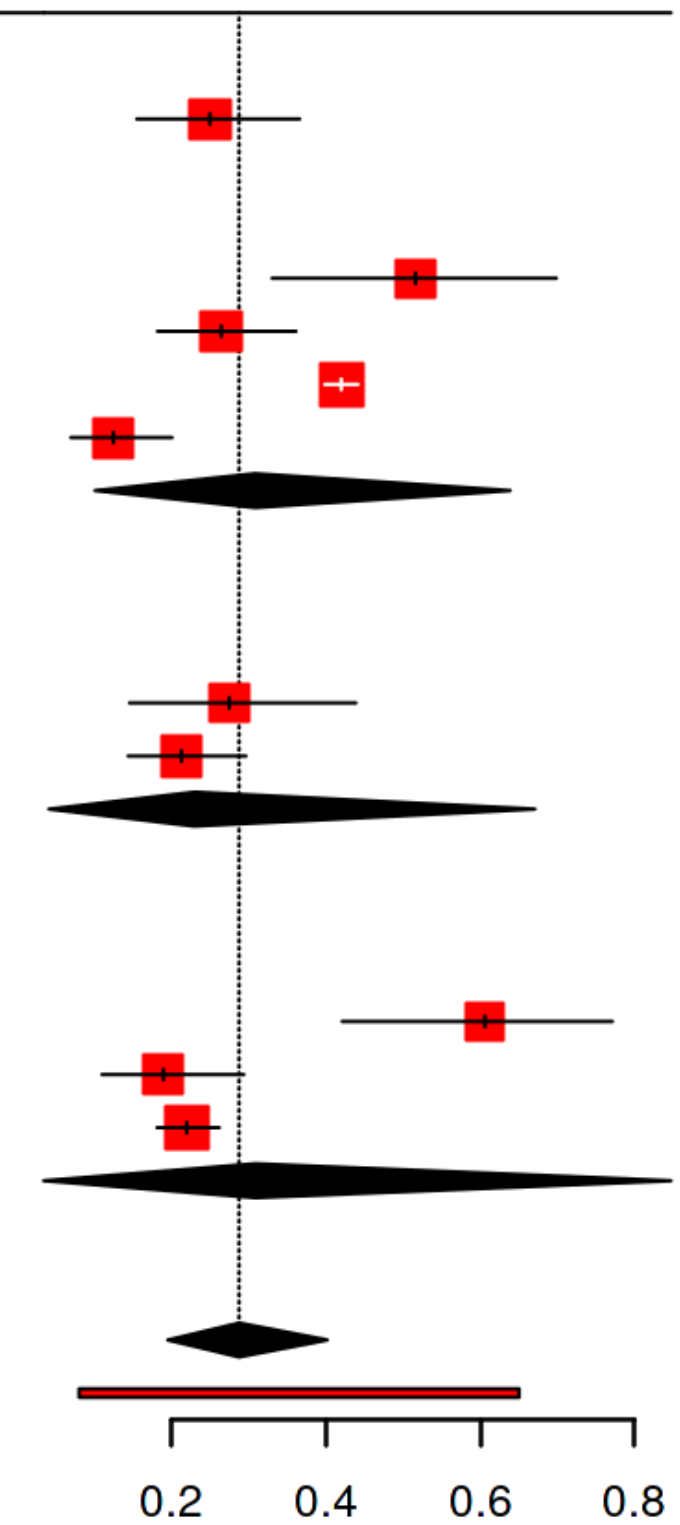

Footnotes:

[1] Ten studies analyzed totaling of 3,087 subjects.

[2] Only last timepoint data was included from studies with multiple timepoints of follow-up. Data from all timepoints can be found in Table S3.

[3] Based on the analysis performed using a random effects model with the inverse variance method and logit transformation, the summarized proportion is 0.29 with a 95% confidence interval of 0.20-0.40.

[4] Significant heterogeneity detected (p<0.01), suggesting inconsistent effects in magnitude and/or direction. The I^2 value indicates that 93% of the variability among studies arises from heterogeneity rather than random chance.

Figure S2d:

Forest Plot: Depression Prevalence in All Studies at ≤6 and ≥12months *without Liao et al.*

Study or Subgroup	Events	Total	Weight	IV, Random, 95% CI
**Subgroup = 0-6 months**				
Quinn 2023	18	72	9.9%	0.25 [0.16; 0.37]
Demir 2008	16	31	8.9%	0.52 [0.33; 0.70]
Meyers 2020	27	102	10.4%	0.26 [0.18; 0.36]
Mikolić 2021	867	2064	11.6%	0.42 [0.40; 0.44]
Wintermann 2023	14	112	9.8%	0.12 [0.07; 0.20]
**Total (95% CI)**		**2381**	**50.6%**	**0.30 [0.14; 0.52]**
Heterogeneity: $Tau^2 = 0.4415$; $Chi^2 = 48.22$, df = 4 (P < 0.0001); $I^2 = 91.7\%$				
**Subgroup = 12+ months**				
Khajavikhan 2016	11	40	9.0%	0.28 [0.15; 0.44]
Sonneville 2023	26	122	10.5%	0.21 [0.14; 0.30]
Geurts 2013	20	33	8.9%	0.61 [0.42; 0.77]
Ragoschke-Schumm 2015	15	79	9.7%	0.19 [0.11; 0.29]
Levine 2014	95	432	11.3%	0.22 [0.18; 0.26]
**Total (95% CI)**		**706**	**49.4%**	**0.28 [0.13; 0.49]**
Heterogeneity: $Tau^2 = 0.2569$; $Chi^2 = 22.53$, df = 4 (P = 0.0002); $I^2 = 82.2\%$				
**Total (95% CI)**		**3087**	**100.0%**	**0.29 [0.20; 0.40]**
**Prediction interval**				**[0.08; 0.65]**

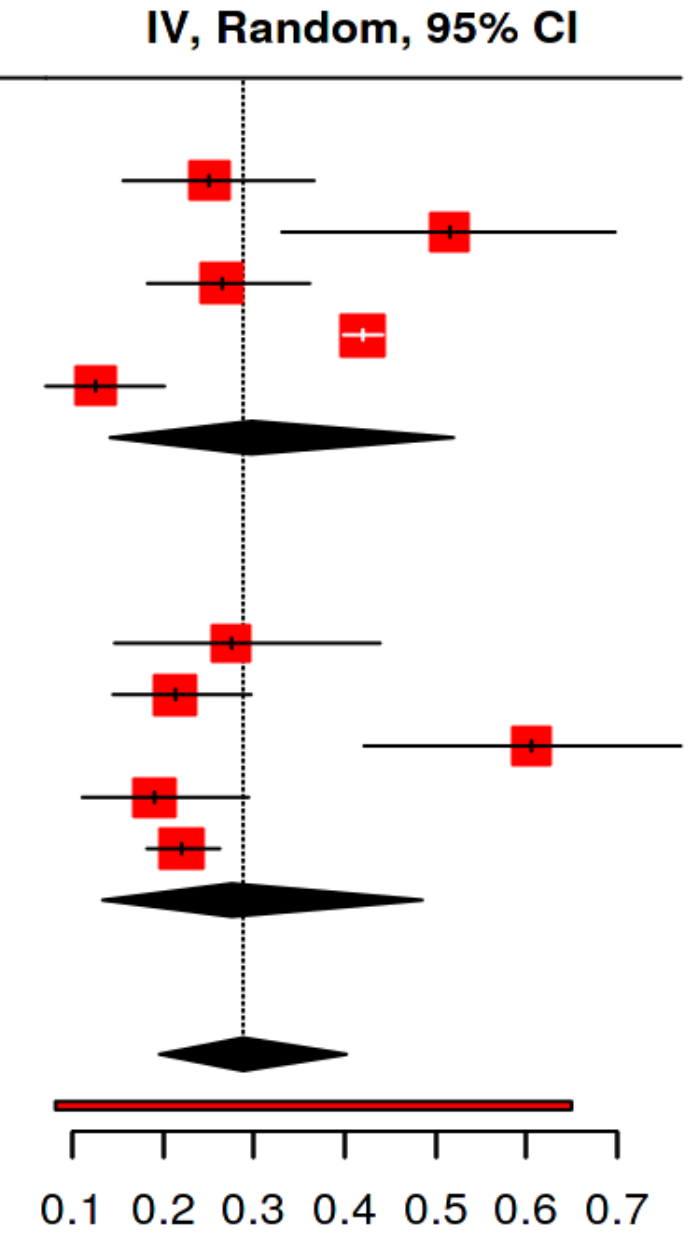

Heterogeneity: $Tau^2 = 0.4078$; $Chi^2 = 129.70$, df = 9 (P < 0.0001); $I^2 = 93.1\%$
Test for subgroup differences: $Chi^2 = 0.05$, df = 1 (P = 0.8257)

Footnotes:

[1] Ten studies analyzed totaling 3,087 subjects.

[2] Only last timepoint data was included from studies with multiple timepoints of follow-up. Data from all timepoints can be found in Table S3.

[3] Based on the analysis performed using a random effects model with the inverse variance method and logit transformation, the summarized proportion is 0.29 with a 95% confidence interval of 0.20-0.40.

[4] Significant heterogeneity detected (p<0.01), suggesting inconsistent effects in magnitude and/or direction. The I^2 value indicates that 93% of the variability among studies arises from heterogeneity rather than random chance.

Figure S2e:

Forest Plot: Depression Prevalence in All Studies *without "Outliers"* at ≤3, 6, 12, ≥18 months

Study or Subgroup	Events	Total	Weight	IV, Random, 95% CI
**Subgroup = 0-3 months**				
Quinn 2023	18	72	12.1%	0.25 [0.16; 0.37]
**Subgroup = 6 months**				
Demir 2008	16	31	9.4%	0.52 [0.33; 0.70]
Meyers 2020	27	102	13.9%	0.26 [0.18; 0.36]
Wintermann 2023	14	112	11.7%	0.12 [0.07; 0.20]
**Total (95% CI)**		**245**	**34.9%**	**0.27 [0.03; 0.81]**
Heterogeneity: Tau^2 = 0.7044; Chi^2 = 19.39, df = 2 (P < 0.0001); I^2 = 89.7%				
**Subgroup = 12 months**				
Khajavikhan 2016	11	40	9.5%	0.28 [0.15; 0.44]
Sonneville 2023	26	122	14.0%	0.21 [0.14; 0.30]
**Total (95% CI)**		**162**	**23.5%**	**0.23 [0.04; 0.67]**
Heterogeneity: Tau^2 = 0; Chi^2 = 0.65, df = 1 (P = 0.4197); I^2 = 0%				
**Subgroup = 18+ months**				
Ragoschke-Schumm 2015	15	79	11.6%	0.19 [0.11; 0.29]
Levine 2014	95	432	17.8%	0.22 [0.18; 0.26]
**Total (95% CI)**		**511**	**29.4%**	**0.22 [0.11; 0.38]**
Heterogeneity: Tau^2 = 0; Chi^2 = 0.36, df = 1 (P = 0.5508); I^2 = 0%				
**Total (95% CI)**		**990**	**100.0%**	**0.24 [0.17; 0.32]**
**Prediction interval**				**[0.11; 0.43]**

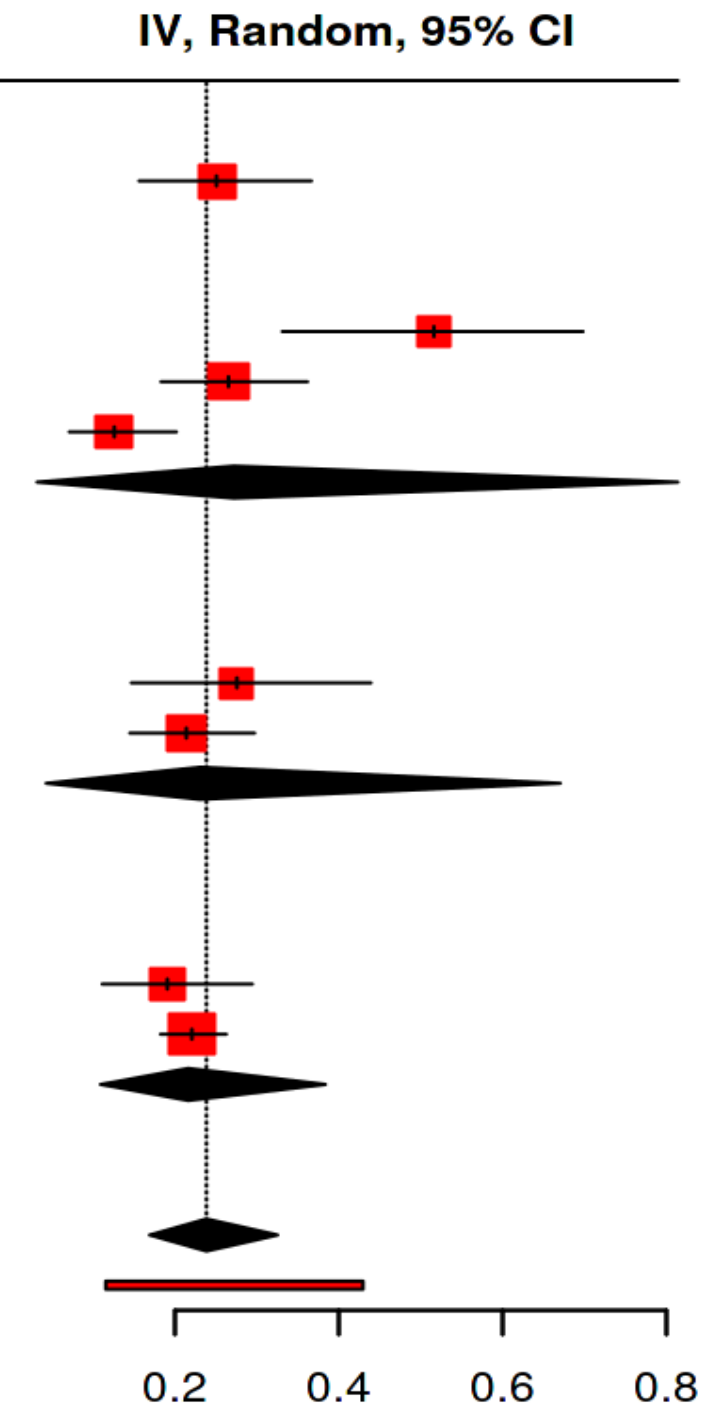

Heterogeneity: Tau^2 = 0.1155; Chi^2 = 21.67, df = 7 (P = 0.0029); I^2 = 67.7%
Test for subgroup differences: Chi^2 = 0.91, df = 3 (P = 0.8241)

Footnotes:

[1] "Outlier" defined as a study having its individual 95%CI outside the overall prevalence 95%CI in Supp Figure S2a. Liao 2020, Geurts 2013, and Mikolic 2021 were removed from this analysis.

[2] Eight studies analyzed totaling 990 subjects.

[3] Based on the analysis performed using a random effects model with the inverse variance method and logit transformation, the summarized proportion is 0.24 with a 95% confidence interval of 0.17-0.32.

[4] Significant heterogeneity detected (p<0.01), suggesting inconsistent effects in magnitude and/or direction. The I^2 value indicates that 68% of the variability among studies arises from heterogeneity rather than random chance.

Figure S2f:

Forest Plot: Depression Prevalence in All Studies *without "Outliers"* at ≤6 and ≥12months

Study or Subgroup	Events	Total	Weight	IV, Random, 95% CI
**Subgroup = 0-6 months**				
Quinn 2023	18	72	12.1%	0.25 [0.16; 0.37]
Demir 2008	16	31	9.4%	0.52 [0.33; 0.70]
Meyers 2020	27	102	13.9%	0.26 [0.18; 0.36]
Wintermann 2023	14	112	11.7%	0.12 [0.07; 0.20]
**Total (95% CI)**		**317**	**47.0%**	**0.26 [0.09; 0.56]**
Heterogeneity: Tau^2 = 0.4245; Chi^2 = 19.39, df = 3 (P = 0.0002); I^2 = 84.5%				
**Subgroup = 12+ months**				
Khajavikhan 2016	11	40	9.5%	0.28 [0.15; 0.44]
Sonneville 2023	26	122	14.0%	0.21 [0.14; 0.30]
Ragoschke-Schumm 2015	15	79	11.6%	0.19 [0.11; 0.29]
Levine 2014	95	432	17.8%	0.22 [0.18; 0.26]
**Total (95% CI)**		**673**	**53.0%**	**0.22 [0.19; 0.25]**
Heterogeneity: Tau^2 = 0; Chi^2 = 1.15, df = 3 (P = 0.7661); I^2 = 0%				
**Total (95% CI)**		**990**	**100.0%**	**0.24 [0.17; 0.32]**
**Prediction interval**				**[0.11; 0.43]**

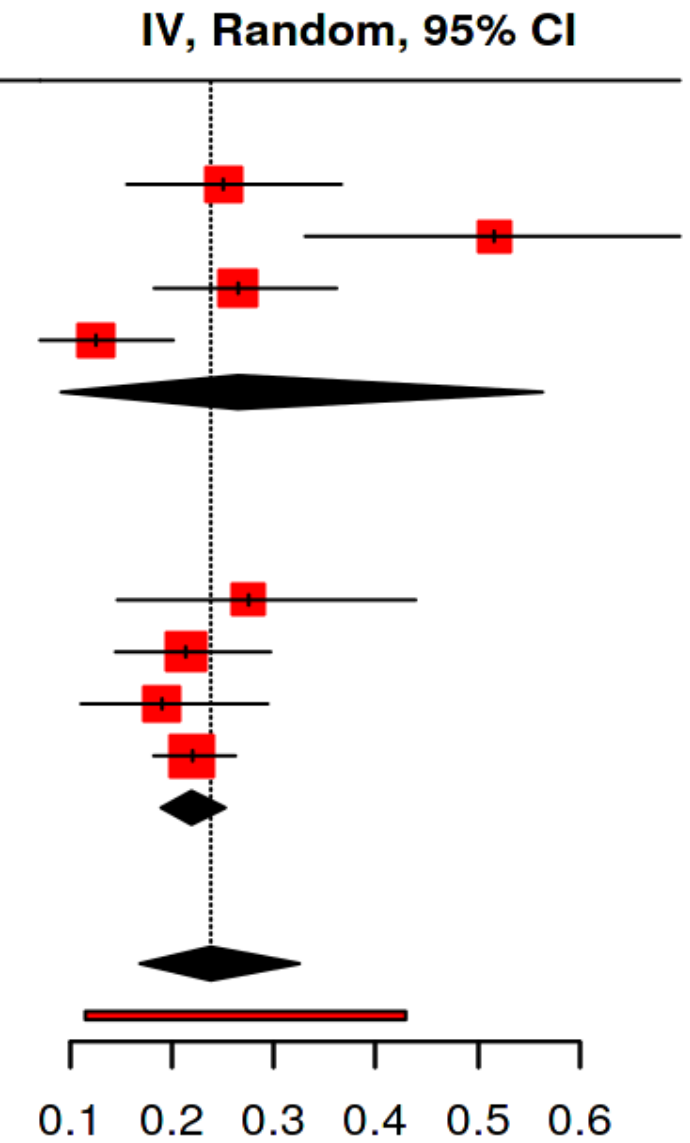

Heterogeneity: Tau^2 = 0.1155; Chi^2 = 21.67, df = 7 (P = 0.0029); I^2 = 67.7%
Test for subgroup differences: Chi^2 = 0.37, df = 1 (P = 0.5412)

Footnotes:

[1] "Outlier" defined as a study having their individual 95%CI outside the overall prevalence 95%CI in Supp Figure S2a. Liao 2020, Geurts 2013, and Mikolic 2021 were removed from this analysis.

[2] Eight studies analyzed totaling 990 subjects.

[3] Based on the analysis performed using a random effects model with the inverse variance method and logit transformation, the summarized proportion is 0.24 with a 95% confidence interval of 0.17-0.32.

[4] Significant heterogeneity detected (p<0.01), suggesting inconsistent effects in magnitude and/or direction. The I^2 value indicates that 68% of the variability among studies arises from heterogeneity rather than random chance.

Supplemental Figure S3:

Figure S3a:

Forest Plot: Depression Prevalence in Studies Using Validated Tools to Measure Depression at ≤3, 6, 12, and ≥18 Months

Study or Subgroup	Events	Total	Weight	IV, Random, 95% CI
**Subgroup = 0-3 months**				
Quinn 2023	18	72	12.2%	0.25 [0.16; 0.37]
**Subgroup = 6 months**				
Meyers 2020	27	102	12.8%	0.26 [0.18; 0.36]
Mikolić 2021	867	2064	14.3%	0.42 [0.40; 0.44]
Wintermann 2023	14	112	12.0%	0.12 [0.07; 0.20]
**Total (95% CI)**		**2278**	**39.1%**	**0.26 [0.04; 0.72]**
Heterogeneity: $Tau^2 = 0.6179$; $Chi^2 = 39.85$, df = 2 ($P < 0.0001$); $I^2 = 95\%$				
**Subgroup = 12 months**				
Khajavikhan 2016	11	40	11.0%	0.28 [0.15; 0.44]
Sonneville 2023	26	122	12.8%	0.21 [0.14; 0.30]
**Total (95% CI)**		**162**	**23.9%**	**0.23 [0.04; 0.67]**
Heterogeneity: $Tau^2 = 0$; $Chi^2 = 0.65$, df = 1 ($P = 0.4197$); $I^2 = 0\%$				
**Subgroup = 18+ months**				
Geurts 2013	20	33	11.0%	0.61 [0.42; 0.77]
Levine 2014	95	432	13.9%	0.22 [0.18; 0.26]
**Total (95% CI)**		**465**	**24.9%**	**0.39 [0.00; 1.00]**
Heterogeneity: $Tau^2 = 1.3697$; $Chi^2 = 20.51$, df = 1 ($P < 0.0001$); $I^2 = 95.1\%$				
**Total (95% CI)**		**2977**	**100.0%**	**0.28 [0.18; 0.41]**
**Prediction interval**				**[0.07; 0.66]**

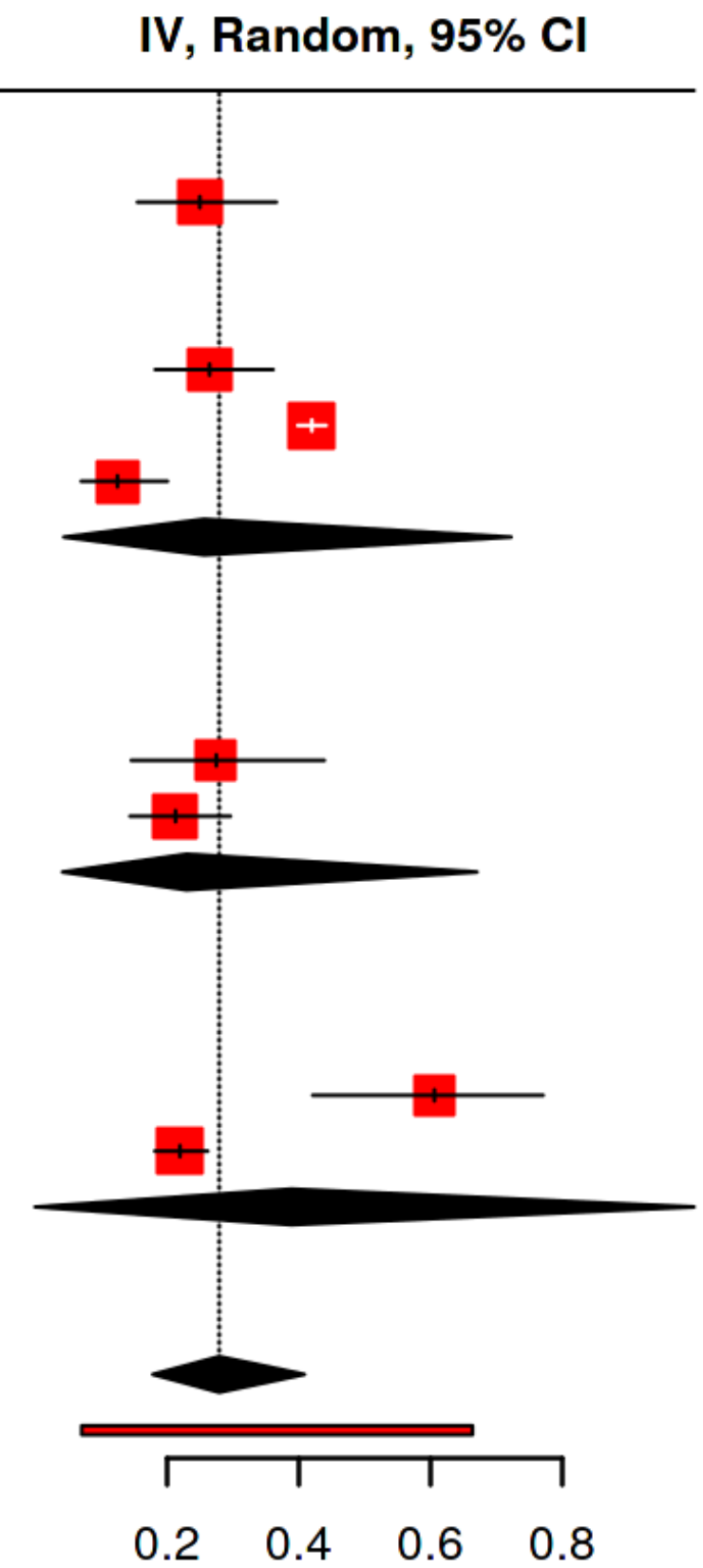

Heterogeneity: $Tau^2 = 0.4141$; $Chi^2 = 116.66$, df = 7 ($P < 0.0001$); $I^2 = 94.0\%$
Test for subgroup differences: $Chi^2 = 0.91$, df = 3 ($P = 0.8234$)

Footnotes:

[1] Eight studies analyzed totaling 2,977 subjects.

[2] Only last timepoint data was included from studies with multiple timepoints of follow-up. Data from all timepoints can be found in Table S3.

[3] Based on the analysis performed using a random effects model with the inverse variance method and logit transformation, the summarized proportion is 0.28 with a 95% confidence interval of 0.18-0.41.

[4] Significant heterogeneity detected ($p<0.01$), suggesting inconsistent effects in magnitude and/or direction. The I^2 value indicates that 94% of the variability among studies arises from heterogeneity rather than random chance.

Figure S3b:

Forest Plot: Depression Prevalence in Studies Using Validated Tools to Measure Depression at ≤6 and ≥12 Months

Study or Subgroup	Events	Total	Weight	IV, Random, 95% CI
**Subgroup = 0-6 months**				
Quinn 2023	18	72	12.2%	0.25 [0.16; 0.37]
Meyers 2020	27	102	12.8%	0.26 [0.18; 0.36]
Mikolić 2021	867	2064	14.3%	0.42 [0.40; 0.44]
Wintermann 2023	14	112	12.0%	0.12 [0.07; 0.20]
**Total (95% CI)**		**2350**	**51.3%**	**0.26 [0.11; 0.50]**
Heterogeneity: $Tau^2 = 0.5058$; $Chi^2 = 46.55$, df = 3 ($P < 0.0001$); $I^2 = 93.6\%$				
**Subgroup = 12+ months**				
Khajavikhan 2016	11	40	11.0%	0.28 [0.15; 0.44]
Sonneville 2023	26	122	12.8%	0.21 [0.14; 0.30]
Geurts 2013	20	33	11.0%	0.61 [0.42; 0.77]
Levine 2014	95	432	13.9%	0.22 [0.18; 0.26]
**Total (95% CI)**		**627**	**48.7%**	**0.30 [0.11; 0.60]**
Heterogeneity: $Tau^2 = 0.3302$; $Chi^2 = 21.41$, df = 3 ($P < 0.0001$); $I^2 = 86\%$				
**Total (95% CI)**		**2977**	**100.0%**	**0.28 [0.18; 0.41]**
**Prediction interval**				**[0.07; 0.66]**

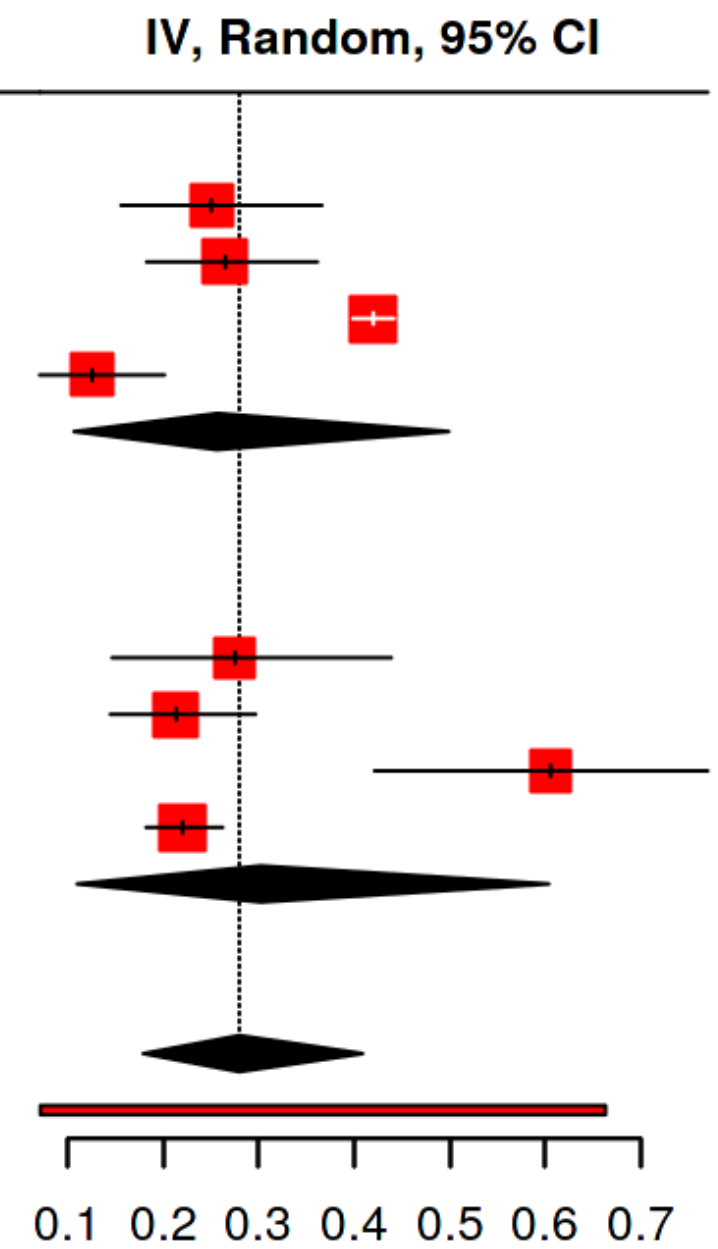

Heterogeneity: $Tau^2 = 0.4141$; $Chi^2 = 116.66$, df = 7 ($P < 0.0001$); $I^2 = 94.0\%$
Test for subgroup differences: $Chi^2 = 0.20$, df = 1 ($P = 0.6571$)

Footnotes:

[1] Eight studies analyzed totaling 2,977 subjects.

[2] Only last timepoint data was included from studies with multiple timepoints of follow-up. Data from all timepoints can be found in Table S3.

[3] Based on the analysis performed using a random effects model with the inverse variance method and logit transformation, the summarized proportion is 0.28 with a 95% confidence interval of 0.18-0.41.

[4] Significant heterogeneity detected (*p*<0.01), suggesting inconsistent effects in magnitude and/or direction. The I^2 value indicates that 94% of the variability among studies arises from heterogeneity rather than random chance.

Figure S3c:

Forest Plot: Depression Prevalence in Studies Using Validated Tools *without "Outliers"* to Measure Depression at ≤3, 6, 12, and ≥18 Months

Study or Subgroup	Events	Total	Weight	IV, Random, 95% CI
**Subgroup = 0-3 months**				
Quinn 2023	18	72	13.7%	0.25 [0.16; 0.37]
**Subgroup = 6 months**				
Meyers 2020	27	102	14.4%	0.26 [0.18; 0.36]
Mikolić 2021	867	2064	16.1%	0.42 [0.40; 0.44]
Wintermann 2023	14	112	13.5%	0.12 [0.07; 0.20]
**Total (95% CI)**		**2278**	**43.9%**	**0.26 [0.04; 0.72]**
Heterogeneity: $Tau^2 = 0.6179$; $Chi^2 = 39.85$, df = 2 (P < 0.0001); $I^2 = 95\%$				
**Subgroup = 12 months**				
Khajavikhan 2016	11	40	12.4%	0.28 [0.15; 0.44]
Sonneville 2023	26	122	14.4%	0.21 [0.14; 0.30]
**Total (95% CI)**		**162**	**26.8%**	**0.23 [0.04; 0.67]**
Heterogeneity: $Tau^2 = 0$; $Chi^2 = 0.65$, df = 1 (P = 0.4197); $I^2 = 0\%$				
**Subgroup = 18+ months**				
Levine 2014	95	432	15.6%	0.22 [0.18; 0.26]
**Total (95% CI)**		**2944**	**100.0%**	**0.25 [0.17; 0.34]**
**Prediction interval**				**[0.06; 0.64]**

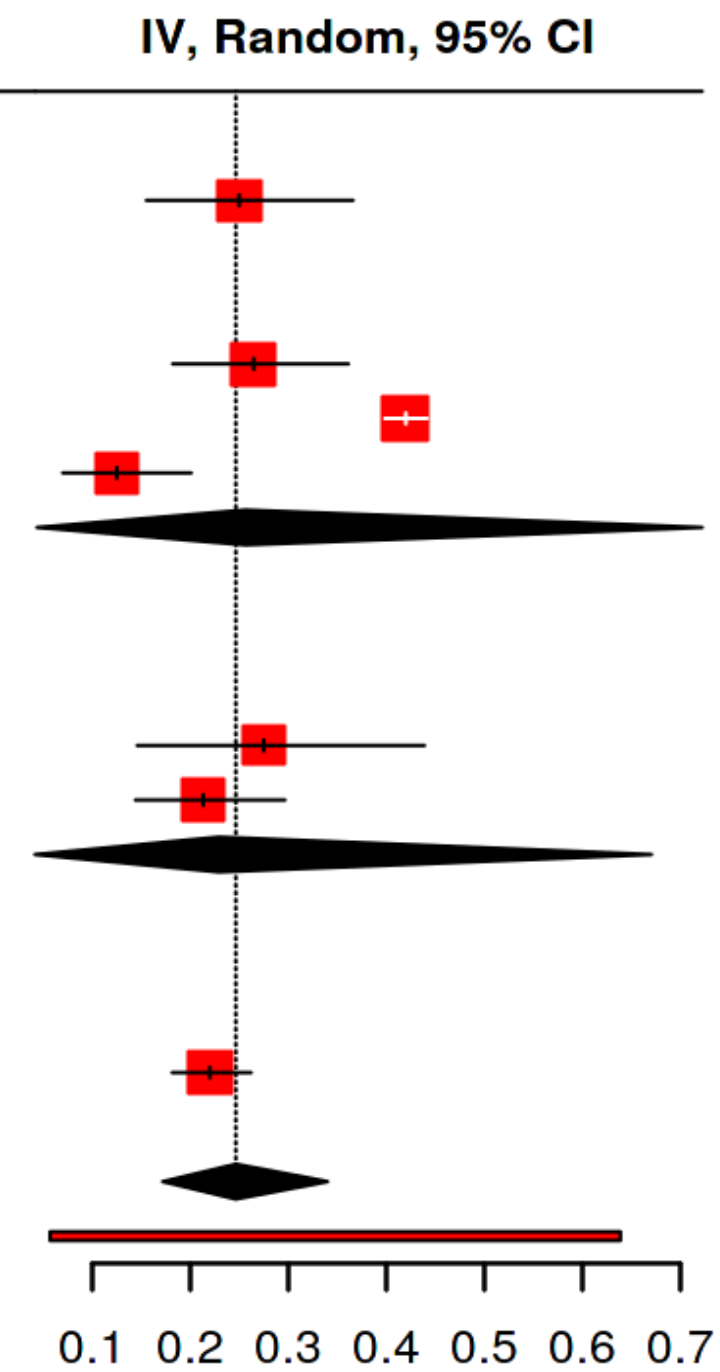

Heterogeneity: $Tau^2 = 0.4097$; $Chi^2 = 109.37$, df = 6 (P < 0.0001); $I^2 = 94.5\%$
Test for subgroup differences: $Chi^2 = 0.47$, df = 3 (P = 0.9262)

Footnotes:

[1] "Outlier" defined as a study having its individual 95%CI outside the overall prevalence 95%CI in Supp Figure S3a. Geurts 2013 was removed from this analysis.

[2] Seven studies analyzed totaling 2,944 subjects.

[3] Based on the analysis performed using a random effects model with the inverse variance method and logit transformation, the summarized proportion is 0.25 with a 95% confidence interval of 0.17-0.34.

[3] Significant heterogeneity detected (p<0.01), suggesting inconsistent effects in magnitude and/or direction. The I^2 value indicates that 94% of the variability among studies arises from heterogeneity rather than random chance.

Figure S3d:

Forest Plot: Depression Prevalence in Studies Using Validated Tools *without "Outliers"* to Measure Depression at ≤6 and ≥12 Months

Study or Subgroup	Events	Total	Weight	IV, Random, 95% CI
**Subgroup = 0-6 months**				
Quinn 2023	18	72	13.7%	0.25 [0.16; 0.37]
Meyers 2020	27	102	14.4%	0.26 [0.18; 0.36]
Mikolić 2021	867	2064	16.1%	0.42 [0.40; 0.44]
Wintermann 2023	14	112	13.5%	0.12 [0.07; 0.20]
**Total (95% CI)**		**2350**	**57.6%**	**0.26 [0.11; 0.50]**
Heterogeneity: $Tau^2 = 0.5058$; $Chi^2 = 46.55$, df = 3 ($P < 0.0001$); $I^2 = 93.6\%$				
**Subgroup = 12+ months**				
Khajavikhan 2016	11	40	12.4%	0.28 [0.15; 0.44]
Sonneville 2023	26	122	14.4%	0.21 [0.14; 0.30]
Levine 2014	95	432	15.6%	0.22 [0.18; 0.26]
**Total (95% CI)**		**594**	**42.4%**	**0.22 [0.18; 0.27]**
Heterogeneity: $Tau^2 = 0$; $Chi^2 = 0.71$, df = 2 ($P = 0.7005$); $I^2 = 0\%$				
**Total (95% CI)**		**2944**	**100.0%**	**0.25 [0.17; 0.34]**
**Prediction interval**				**[0.06; 0.64]**

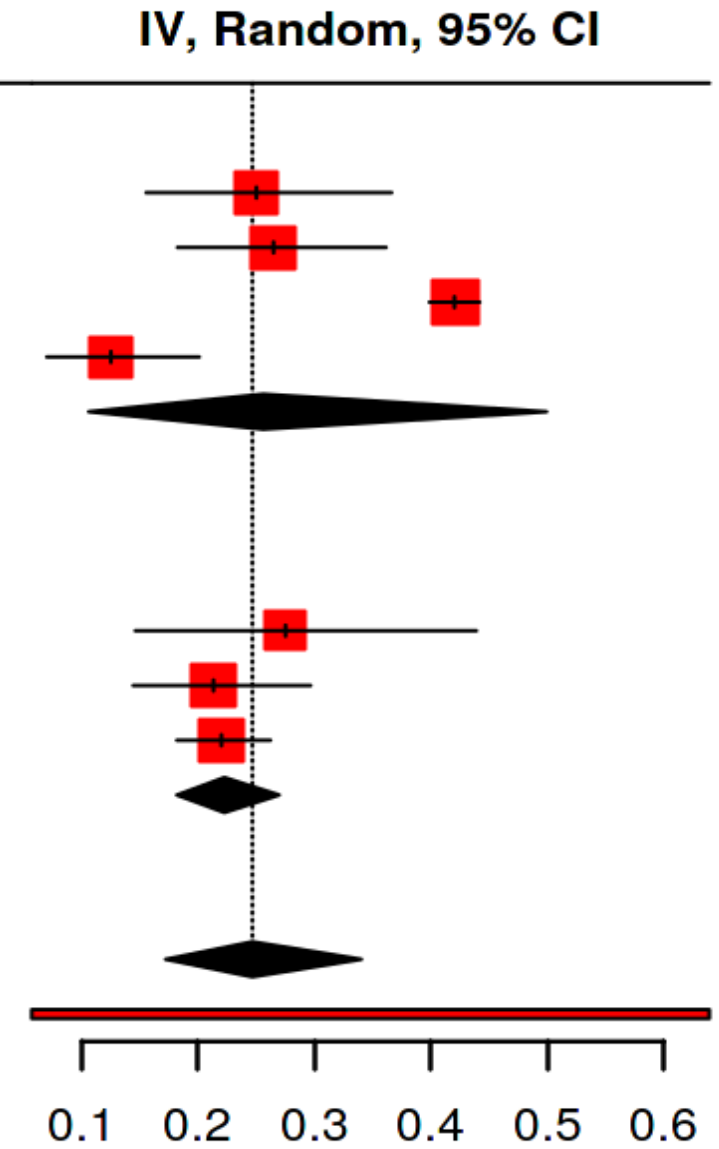

Heterogeneity: $Tau^2 = 0.4097$; $Chi^2 = 109.37$, df = 6 ($P < 0.0001$); $I^2 = 94.5\%$
Test for subgroup differences: $Chi^2 = 0.29$, df = 1 ($P = 0.5884$)

Footnotes:

[1] "Outlier" defined as a study having its individual 95%CI outside the overall prevalence 95%CI in Supp Figure S3a. Geurts 2013 was removed from this analysis.

[2] Seven studies analyzed totaling 2,944 subjects.

[3] Based on the analysis performed using a random effects model with the inverse variance method and logit transformation, the summarized proportion is 0.25 with a 95% confidence interval of 0.17-0.34.

[3] Significant heterogeneity detected (p<0.01), suggesting inconsistent effects in magnitude and/or direction. The I^2 value indicates that 94% of the variability among studies arises from heterogeneity rather than random chance.

Supplemental Figure S4:

Forest Plot: Pooled HADS-D Scores for Depression Symptoms at ≤3, 6, 12, and ≥18 Months

Study or Subgroup	Mean	SD	Total	Weight	Mean IV, Random, 95% CI
**subgroup = 0-3 months**					
Sadlonova 2021	4.40	4.4000	360	15.9%	4.40 [3.95; 4.85]
Quinn 2023	4.50	4.1000	72	14.4%	4.50 [3.55; 5.45]
Bannon 2020	8.60	2.9000	16	12.5%	8.60 [7.18; 10.02]
Bannon 2022	5.70	4.2000	49	13.6%	5.70 [4.52; 6.88]
**Total (95% CI)**			**497**	**56.5%**	**5.69 [4.16; 7.22]**
Heterogeneity: $Tau^2 = 2.1562$; $Chi^2 = 33.05$, df = 3 ($P < 0.0001$); $I^2 = 90.9\%$					
**subgroup = 6 months**					
Meyers 2020	4.80	5.4000	102	14.1%	4.80 [3.75; 5.85]
**subgroup = 12 months**					
Sonneville 2023	7.33	5.2000	122	14.5%	7.33 [6.41; 8.25]
**subgroup = 18+ months**					
Azouvi 2016	5.20	4.5000	118	14.9%	5.20 [4.39; 6.01]
**Total (95% CI)**			**839**	**100.0%**	**5.72 [4.69; 6.75]**
**Prediction interval**					**[2.30; 9.14]**

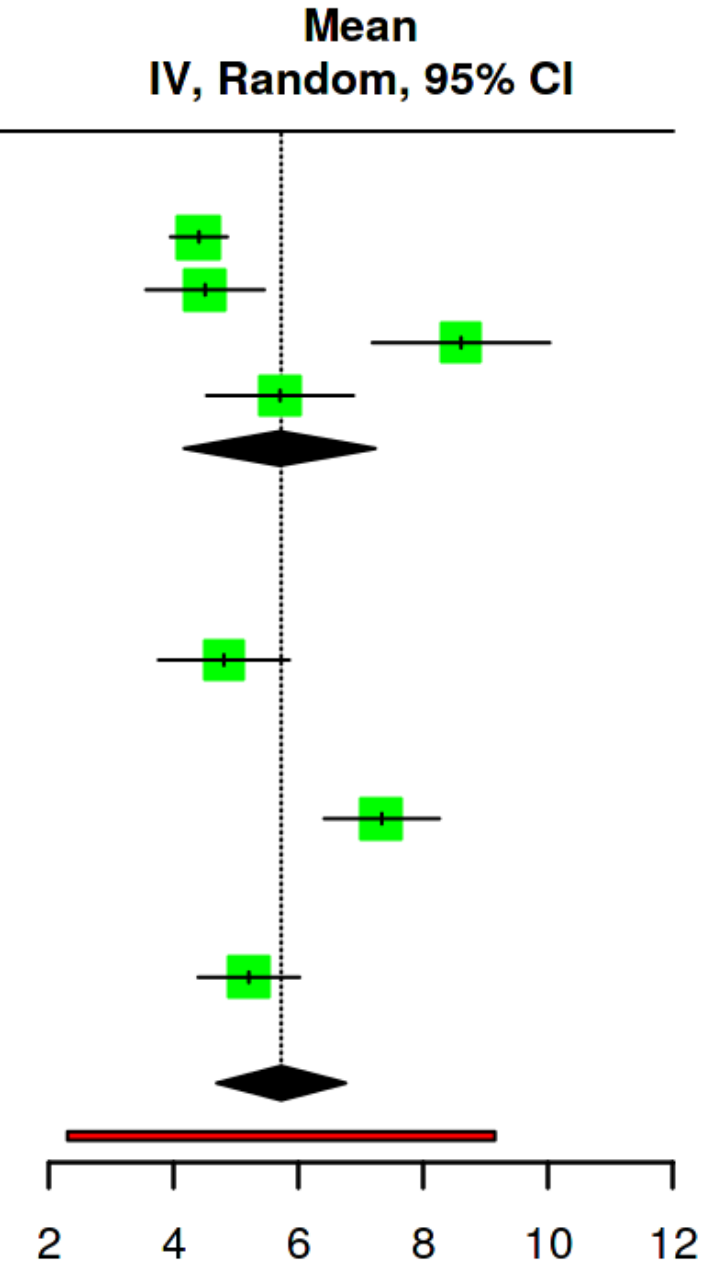

Heterogeneity: $Tau^2 = 1.6810$; $Chi^2 = 57.64$, df = 6 ($P < 0.0001$); $I^2 = 89.6\%$
Test for subgroup differences: $Chi^2 = 16.17$, df = 3 ($P = 0.0010$)

Footnotes:

[1] Seven studies analyzed totaling 839 subjects.

[2] Based on the analysis performed using random effects model with inverse variance method, the summarized raw means (MRAW) is 5.72 with a 95% confidence interval of 4.69-6.75.

[3] Significant heterogeneity detected (*p*<0.01), suggesting inconsistent effects in magnitude and/or direction. The I^2 value indicates that 90% of the variability among studies arises from heterogeneity rather than random chance.

[4] Only last timepoint data was included from studies with multiple timepoints of follow-up. Data from all timepoints can be found in Table S3.

[5] All studies used HADS-D cutoff of ≥7 or ≥8 for depression.

[6] Sonneville mean (SD) calculated from a median (IQR) of 7 (4,11).

Supplemental Table S4: Characteristics of manuscripts reporting on anxiety outcomes					
**Citation**	**Instrument Used**	**Study *N* at Follow-Up**	**Follow-Up Timeline (Mo)**	**Anxiety Prevalence (%)**	**Anxiety Score Mean (SD)**
Meyers 2020	HADS-A	102	0<br>3<br>6	40<br>40<br>36	6.95 (4.83)<br>6.24 (5.16)<br>6.37 (5.73)
Bannon 2022	HADS-A	58<br>53<br>49	0<br>1.5<br>6	NR	8.7 (5.0)<br>5.9 (4.7)<br>6.8 (5.4)
Sadlonova 2021	HADS-A	356	0	13.2	5.3 (4.4)
Bannon 2020	HADS-A	16<br>16<br>14	0<br>1.5<br>6	66.7<br>77.8<br>55.6 [a]	10.2 (4.9)<br>12.7 (5.9)<br>9.9 (6.5)
Azouvi 2016	HADS-A	118	48	NR	7.2 (4.5) [b]
Khajavikhan 2016	HADS-A	40	12	37.5	NR
Quinn 2023	HADS-A	72	0	42	6.8 (4.6)
Sonneville 2023	HADS-A	174	12	49	7 (5-11) [c]
Diaz 2012	HADS	33	18.4	NR	10.5 (0.9) [d]
Mikolić 2021	GAD-7	2059	6	14.8	NR
Zhao 2021	SAS	99	3	NR	37.9 (12.9)

Abbreviations: HADS-A: Hospital Anxiety and Depression Scale, Anxiety Subscale; HADS: Hospital Anxiety and Depression Scale; GAD-7: General Anxiety Disorder-7; NR: not reported; SAS: Zung Self-Rating Anxiety Scale

[a] Anxiety prevalence here is reported at 3 months rather than 6-month follow-up.

[b] Based on personal communication with author.

[c] Median (IQR) reported within manuscript.

[d] This score represents the mean of combined anxiety and depression scales rather than anxiety alone.

Supplemental Figure S5:

Figure S5a:

Forest Plot: Anxiety Prevalence in Studies Using HADS-A at ≤3, 6, and ≥12 Months

Study or Subgroup	Events	Total	Weight	IV, Random, 95% CI
**Subgroup = 0-3 months**				
Sadlonova 2021	47	356	17.9%	0.13 [0.10; 0.17]
Quinn 2023	30	72	17.2%	0.42 [0.30; 0.54]
**Total (95% CI)**		**428**	**35.1%**	**0.25 [0.00; 1.00]**
Heterogeneity: $Tau^2 = 1.1553$; $Chi^2 = 29.3$, df = 1 ($P < 0.0001$); $I^2 = 96.6\%$				
**Subgroup = 6 months**				
Bannon 2020	8	14	13.2%	0.57 [0.29; 0.82]
Meyers 2020	37	102	17.5%	0.36 [0.27; 0.46]
**Total (95% CI)**		**116**	**30.8%**	**0.43 [0.00; 0.99]**
Heterogeneity: $Tau^2 = 0.1952$; $Chi^2 = 2.17$, df = 1 ($P = 0.1409$); $I^2 = 53.9\%$				
**Subgroup = 12 months**				
Khajavikhan 2016	15	40	16.2%	0.38 [0.23; 0.54]
Sonneville 2023	85	174	18.0%	0.49 [0.41; 0.57]
**Total (95% CI)**		**214**	**34.1%**	**0.45 [0.05; 0.93]**
Heterogeneity: $Tau^2 = 0.0432$; $Chi^2 = 1.67$, df = 1 ($P = 0.1967$); $I^2 = 40\%$				
**Total (95% CI)**		**758**	**100.0%**	**0.37 [0.21; 0.56]**
**Prediction interval**				**[0.05; 0.86]**

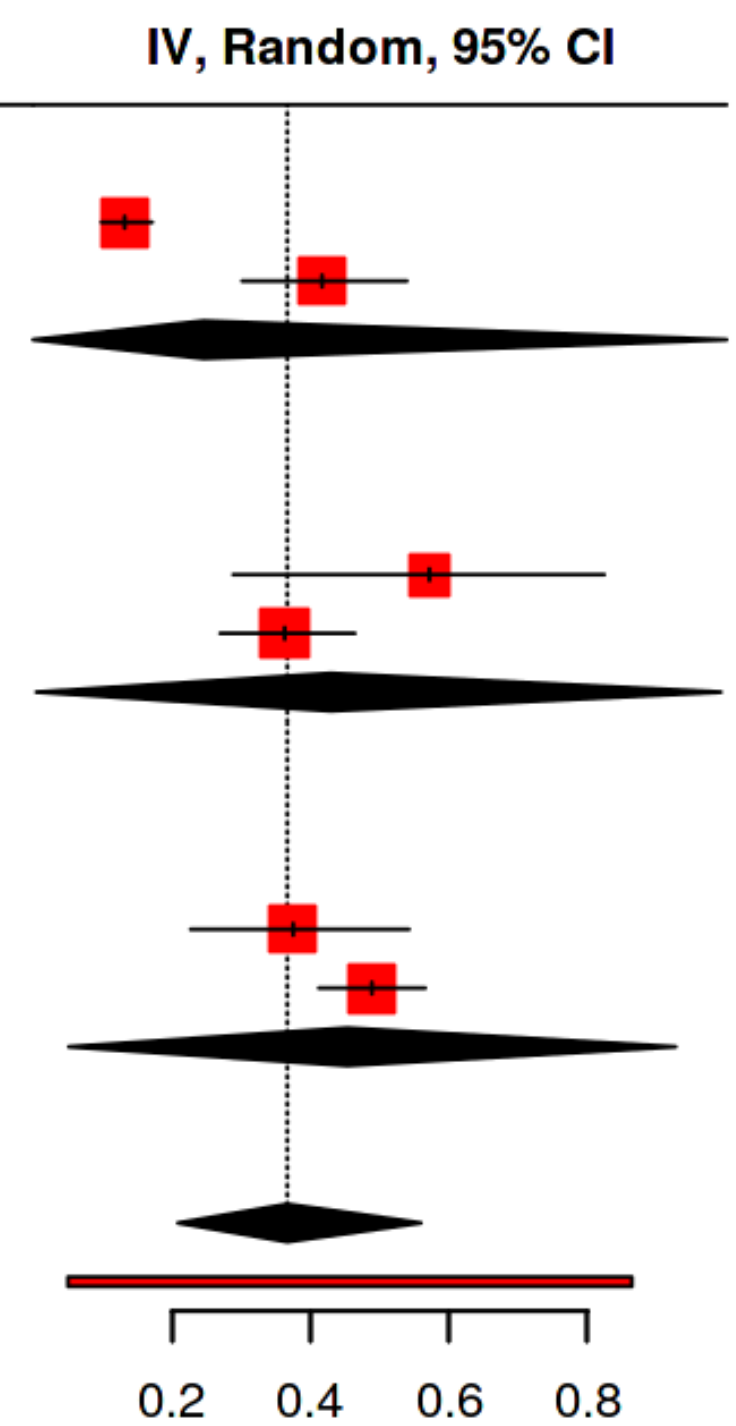

Heterogeneity: $Tau^2 = 0.7297$; $Chi^2 = 81.95$, df = 5 ($P < 0.0001$); $I^2 = 93.9\%$
Test for subgroup differences: $Chi^2 = 1.35$, df = 2 ($P = 0.5083$)

Footnotes:
[1] Six studies analyzed totaling 758 subjects.
[2] Quinn 2023 utilized a HADS-A cutoff of ≥7; all others used ≥8.
[3] Only last timepoint data was included from studies with multiple timepoints of follow-up. Data from all timepoints can be found in Table S4.
[4] Based on the analysis performed using a random effects model with the inverse variance method and logit transformation, the summarized proportion is 0.37 with a 95% confidence interval of 0.21-0.56.
[5] Significant heterogeneity detected (p<0.01), suggesting inconsistent effects in magnitude and/or direction. The I^2 value indicates that 94% of the variability among studies arises from heterogeneity rather than random chance.

Figure S5b:

Forest Plot: Anxiety Prevalence in Studies Using HADS-A at ≤6 and ≥12 Months

Study or Subgroup	Events	Total	Weight	IV, Random, 95% CI
**Subgroup = 0-6 months**				
Sadlonova 2021	47	356	17.9%	0.13 [0.10; 0.17]
Quinn 2023	30	72	17.2%	0.42 [0.30; 0.54]
Bannon 2020	8	14	13.2%	0.57 [0.29; 0.82]
Meyers 2020	37	102	17.5%	0.36 [0.27; 0.46]
**Total (95% CI)**		**544**	**65.9%**	**0.33 [0.11; 0.68]**
Heterogeneity: $Tau^2 = 0.8209$; $Chi^2 = 48.6$, df = 3 ($P < 0.0001$); $I^2 = 93.8\%$				
**Subgroup = 12 months**				
Khajavikhan 2016	15	40	16.2%	0.38 [0.23; 0.54]
Sonneville 2023	85	174	18.0%	0.49 [0.41; 0.57]
**Total (95% CI)**		**214**	**34.1%**	**0.45 [0.05; 0.93]**
Heterogeneity: $Tau^2 = 0.0432$; $Chi^2 = 1.67$, df = 1 ($P = 0.1967$); $I^2 = 40\%$				
**Total (95% CI)**		**758**	**100.0%**	**0.37 [0.21; 0.56]**
**Prediction interval**				**[0.05; 0.86]**

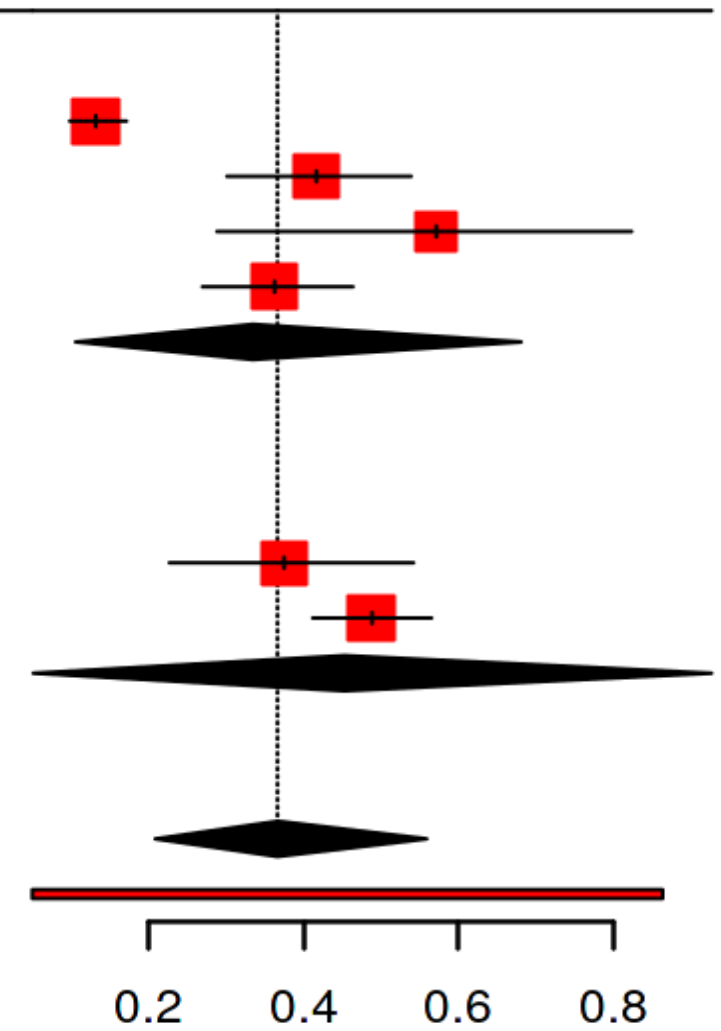

Heterogeneity: $Tau^2 = 0.7297$; $Chi^2 = 81.95$, df = 5 ($P < 0.0001$); $I^2 = 93.9\%$
Test for subgroup differences: $Chi^2 = 0.98$, df = 1 ($P = 0.3211$)

Footnotes:

[1] Six studies analyzed totaling 758 subjects.
[2] Quinn 2023 utilized a HADS-A cutoff of ≥7; all others used ≥8.
[3] Only last timepoint data was included from studies with multiple timepoints of follow-up. Data from all timepoints can be found in Table S4.
[4] Based on the analysis performed using a random effects model with the inverse variance method and logit transformation, the summarized proportion is 0.37 with a 95% confidence interval of 0.21-0.56.
[5] Significant heterogeneity detected (*p*<0.01), suggesting inconsistent effects in magnitude and/or direction. The I^2 value indicates that 94% of the variability among studies arises from heterogeneity rather than random chance.

Supplemental Figure S6:

Figure S6a:

Forest Plot: Anxiety Prevalence in All Studies at ≤3, 6, and ≥12 Months

Study or Subgroup	Events	Total	Weight	IV, Random, 95% CI
**Subgroup = 0-3 months**				
Sadlonova 2021	47	356	15.1%	0.13 [0.10; 0.17]
Quinn 2023	30	72	14.5%	0.42 [0.30; 0.54]
**Total (95% CI)**		**428**	**29.6%**	**0.25 [0.00; 1.00]**
Heterogeneity: Tau^2 = 1.1553; Chi^2 = 29.3, df = 1 (P < 0.0001); I^2 = 96.6%				
**Subgroup = 6 months**				
Bannon 2020	8	14	11.3%	0.57 [0.29; 0.82]
Meyers 2020	37	102	14.8%	0.36 [0.27; 0.46]
Mikolić 2021	305	2059	15.5%	0.15 [0.13; 0.16]
**Total (95% CI)**		**2175**	**41.6%**	**0.32 [0.04; 0.85]**
Heterogeneity: Tau^2 = 0.8310; Chi^2 = 43.13, df = 2 (P < 0.0001); I^2 = 95.4%				
**Subgroup = 12 months**				
Khajavikhan 2016	15	40	13.7%	0.38 [0.23; 0.54]
Sonneville 2023	85	174	15.1%	0.49 [0.41; 0.57]
**Total (95% CI)**		**214**	**28.8%**	**0.45 [0.05; 0.93]**
Heterogeneity: Tau^2 = 0.0432; Chi^2 = 1.67, df = 1 (P = 0.1967); I^2 = 40%				
**Total (95% CI)**		**2817**	**100.0%**	**0.32 [0.18; 0.51]**
**Prediction interval**				**[0.05; 0.83]**

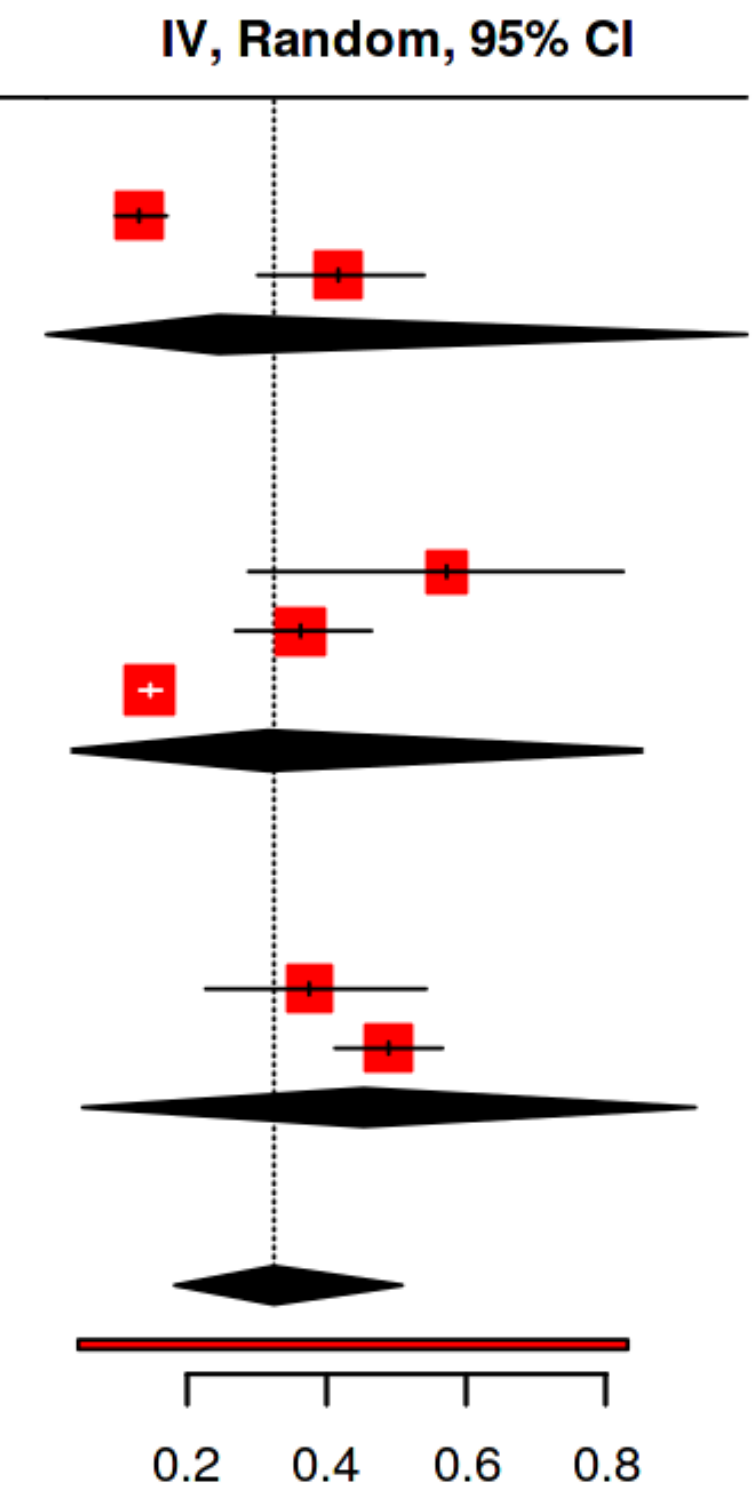

Heterogeneity: Tau^2 = 0.7761; Chi^2 = 174.41, df = 6 (P < 0.0001); I^2 = 96.6%
Test for subgroup differences: Chi^2 = 2.04, df = 2 (P = 0.3608)

Footnotes:
[1] Seven studies analyzed totaling 2,817 subjects.
[2] Only last timepoint data was included from studies with multiple timepoints of follow-up. Data from all timepoints can be found in Table S4.
[3] Based on the analysis performed using a random effects model with the inverse variance method and logit transformation, the summarized proportion is 0.32 with a 95% confidence interval of 0.18-0.51.
[4] Significant heterogeneity detected (p<0.01), suggesting inconsistent effects in magnitude and/or direction. The I^2 value indicates that 97% of the variability among studies arises from heterogeneity rather than random chance.
[5] All studies evaluating anxiety utilized validated instruments (e.g., HADS-A, GAD-7, SAS).

Figure S6b:

Forest Plot: Anxiety Prevalence in All Studies at ≤6 and ≥12 Months

Study or Subgroup	Events	Total	Weight	IV, Random, 95% CI
**Subgroup = 0-6 months**				
Sadlonova 2021	47	356	15.1%	0.13 [0.10; 0.17]
Quinn 2023	30	72	14.5%	0.42 [0.30; 0.54]
Bannon 2020	8	14	11.3%	0.57 [0.29; 0.82]
Meyers 2020	37	102	14.8%	0.36 [0.27; 0.46]
Mikolić 2021	305	2059	15.5%	0.15 [0.13; 0.16]
**Total (95% CI)**		**2603**	**71.2%**	**0.28 [0.11; 0.54]**
Heterogeneity: $Tau^2 = 0.4961$; $Chi^2 = 74.53$, df = 4 ($P < 0.0001$); $I^2 = 94.6\%$				
**Subgroup = 12 months**				
Khajavikhan 2016	15	40	13.7%	0.38 [0.23; 0.54]
Sonneville 2023	85	174	15.1%	0.49 [0.41; 0.57]
**Total (95% CI)**		**214**	**28.8%**	**0.45 [0.05; 0.93]**
Heterogeneity: $Tau^2 = 0.0432$; $Chi^2 = 1.67$, df = 1 ($P = 0.1967$); $I^2 = 40\%$				
**Total (95% CI)**		**2817**	**100.0%**	**0.32 [0.18; 0.51]**
**Prediction interval**				**[0.05; 0.83]**

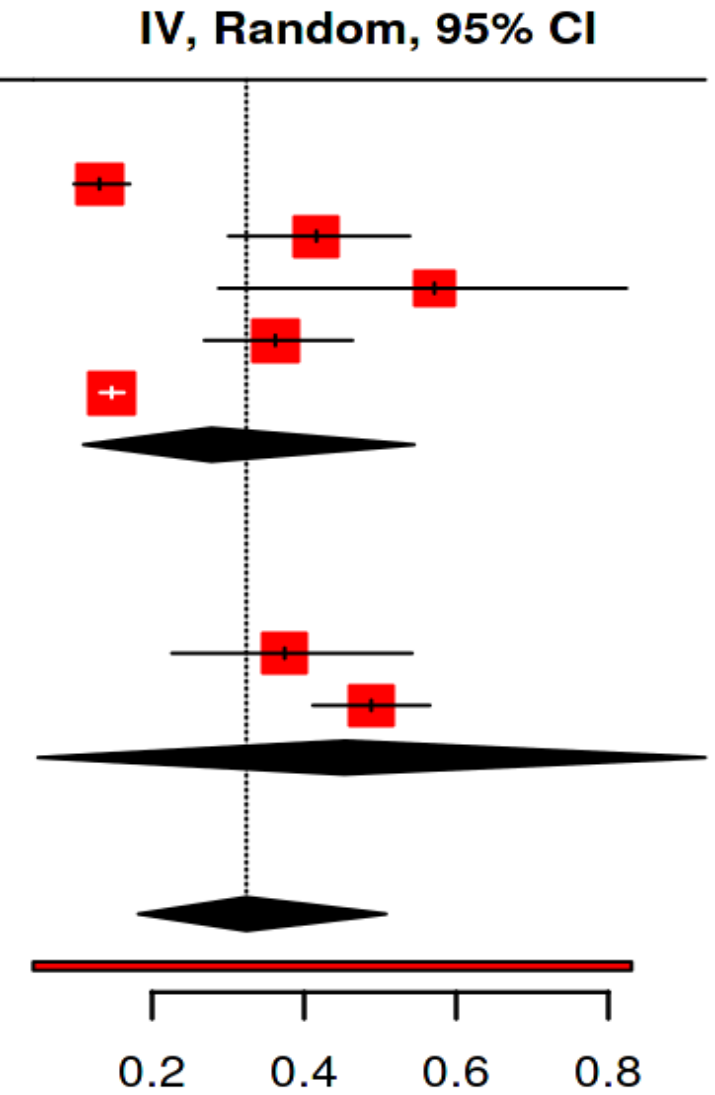

Heterogeneity: $Tau^2 = 0.7761$; $Chi^2 = 174.41$, df = 6 ($P < 0.0001$); $I^2 = 96.6\%$
Test for subgroup differences: $Chi^2 = 2.74$, df = 1 ($P = 0.0978$)

Footnotes:

[1] Seven studies analyzed totaling 2,817 subjects.
[2] Only last timepoint data was included from studies with multiple timepoints of follow-up. Data from all timepoints can be found in Table S4.
[3] Based on the analysis performed using a random effects model with the inverse variance method and logit transformation, the summarized proportion is 0.32 with a 95% confidence interval of 0.18-0.51.
[4] Significant heterogeneity detected (p<0.01), suggesting inconsistent effects in magnitude and/or direction. The I^2 value indicates that 97% of the variability among studies arises from heterogeneity rather than random chance.
[5] All studies evaluating anxiety utilized validated instruments (e.g., HADS-A, GAD-7, SAS).

Figure S6c:

Forest Plot: Anxiety Prevalence in All Studies *without "Outliers"* at ≤3, 6, and ≥12 Months

Study or Subgroup	Events	Total	Weight	IV, Random, 95% CI
**Subgroup = 0-3 months**				
Quinn 2023	30	72	20.9%	0.42 [0.30; 0.54]
**Subgroup = 6 months**				
Bannon 2020	8	14	5.6%	0.57 [0.29; 0.82]
Meyers 2020	37	102	25.3%	0.36 [0.27; 0.46]
**Total (95% CI)**		**116**	**30.9%**	**0.43 [0.00; 0.99]**
Heterogeneity: $Tau^2 = 0.1952$; $Chi^2 = 2.17$, df = 1 (P = 0.1409); $I^2 = 53.9\%$				
**Subgroup = 12 months**				
Khajavikhan 2016	15	40	13.2%	0.38 [0.23; 0.54]
Sonneville 2023	85	174	35.0%	0.49 [0.41; 0.57]
**Total (95% CI)**		**214**	**48.2%**	**0.45 [0.05; 0.93]**
Heterogeneity: $Tau^2 = 0.0432$; $Chi^2 = 1.67$, df = 1 (P = 0.1967); $I^2 = 40\%$				
**Total (95% CI)**		**402**	**100.0%**	**0.43 [0.35; 0.52]**
**Prediction interval**				**[0.29; 0.58]**

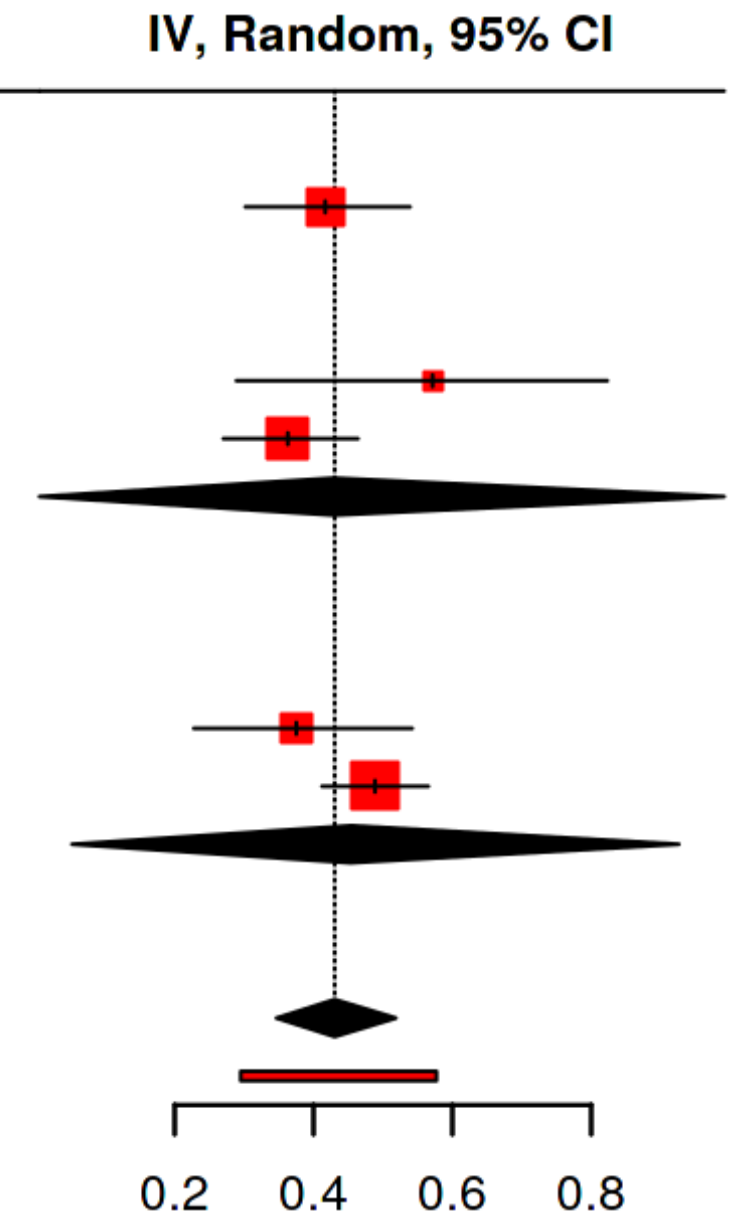

Heterogeneity: $Tau^2 = 0.0277$; $Chi^2 = 5.88$, df = 4 (P = 0.2081); $I^2 = 32.0\%$
Test for subgroup differences: $Chi^2 = 0.22$, df = 2 (P = 0.8969)

Footnotes:

1 "Outlier" defined as a study having its individual 95%CI outside the overall prevalence 95%CI in Supp Figure S6a. Sadlonova 2021 and Mikolic 2021 were removed from this analysis.

2 Five studies using validated instruments analyzed totaling 402 subjects.

3 Only last timepoint data was included from studies with multiple timepoints of follow-up. Data from all timepoints can be found in Table S4.

4 Based on the analysis performed using a random effects model with the inverse variance method and logit transformation, the summarized proportion is 0.43 with a 95% confidence interval of 0.35-0.52.

5 Significant heterogeneity was not observed, indicating that effect sizes across studies are consistent in both magnitude and direction.

Figure S6d:

Forest Plot: Anxiety Prevalence in All Studies *without "Outliers"* at ≤6 and ≥12 Months

Study or Subgroup	Events	Total	Weight	IV, Random, 95% CI
**Subgroup = 0-6 months**				
Quinn 2023	30	72	20.9%	0.42 [0.30; 0.54]
Bannon 2020	8	14	5.6%	0.57 [0.29; 0.82]
Meyers 2020	37	102	25.3%	0.36 [0.27; 0.46]
**Total (95% CI)**		**188**	**51.8%**	**0.40 [0.24; 0.58]**
Heterogeneity: $Tau^2 = 0.0128$; $Chi^2 = 2.32$, df = 2 (P = 0.3137); $I^2 = 13.7\%$				
**Subgroup = 12 months**				
Khajavikhan 2016	15	40	13.2%	0.38 [0.23; 0.54]
Sonneville 2023	85	174	35.0%	0.49 [0.41; 0.57]
**Total (95% CI)**		**214**	**48.2%**	**0.45 [0.05; 0.93]**
Heterogeneity: $Tau^2 = 0.0432$; $Chi^2 = 1.67$, df = 1 (P = 0.1967); $I^2 = 40\%$				
**Total (95% CI)**		**402**	**100.0%**	**0.43 [0.35; 0.52]**
**Prediction interval**				**[0.29; 0.58]**

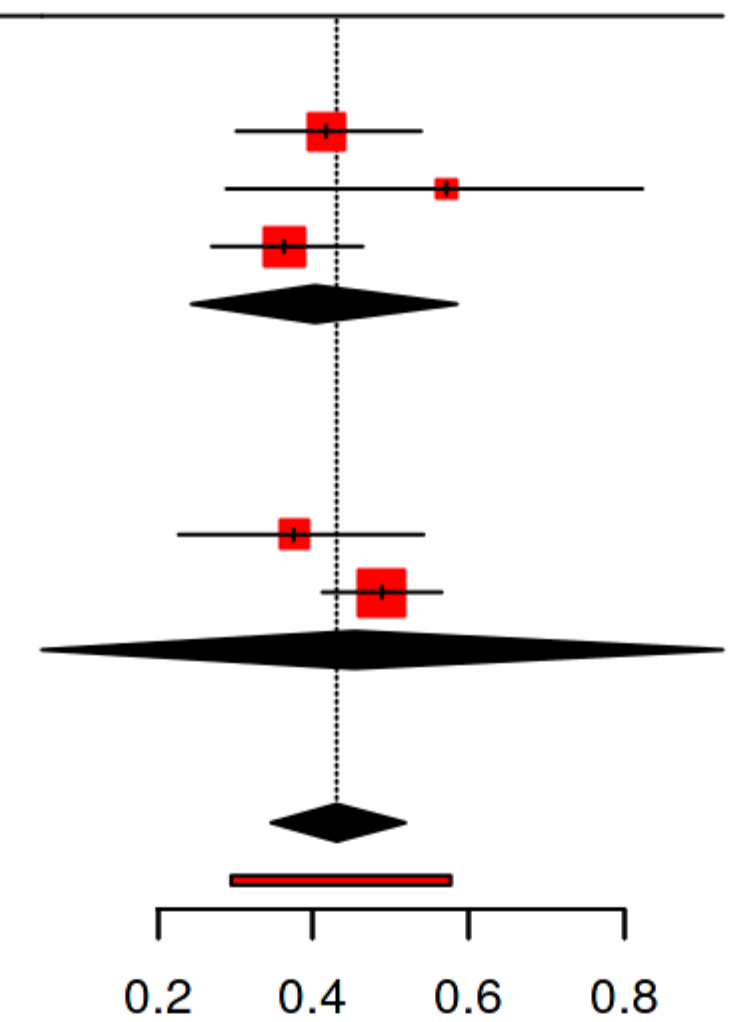

Heterogeneity: $Tau^2 = 0.0277$; $Chi^2 = 5.88$, df = 4 (P = 0.2081); $I^2 = 32.0\%$
Test for subgroup differences: $Chi^2 = 0.56$, df = 1 (P = 0.4531)

Footnotes:

[1] "Outlier" defined as a study having its individual 95%CI outside the overall prevalence 95%CI in Supp Figure S6a. Sadlonova 2021 and Mikolic 2021 were removed from this analysis.

[2] Five studies using validated instruments analyzed totaling 402 subjects.

[3] Only last timepoint data was included from studies with multiple timepoints of follow-up. Data from all timepoints can be found in Table S4.

[4] Based on the analysis performed using a random effects model with the inverse variance method and logit transformation, the summarized proportion is 0.43 with a 95% confidence interval of 0.35-0.52.

[5] Significant heterogeneity not observed, indicating that effect sizes across studies are consistent in both magnitude and direction.

Supplemental Figure S7:

Forest Plot: Pooled HADS-A Scores for Anxiety Symptoms

Study or Subgroup	Mean	SD	Total	Weight	Mean IV, Random, 95% CI
**subgroup = 0-3 months**					
Sadlonova 2021	5.30	4.4000	356	18.9%	5.30 [4.84; 5.76]
Quinn 2023	6.80	4.6000	72	15.1%	6.80 [5.74; 7.86]
**Total (95% CI)**			**428**	**34.0%**	**5.97 [4.51; 7.43]**
Heterogeneity: $Tau^2 = 0.9509$; $Chi^2 = 6.46$, df = 1 (P = 0.0110); $I^2 = 84.5\%$					
**subgroup = 6 months**					
Bannon 2020	9.90	6.5000	14	4.6%	9.90 [6.50; 13.30]
Meyers 2020	6.37	5.7300	102	14.7%	6.37 [5.26; 7.48]
Bannon 2022	6.80	5.4000	49	12.0%	6.80 [5.29; 8.31]
**Total (95% CI)**			**165**	**31.3%**	**6.99 [5.64; 8.35]**
Heterogeneity: $Tau^2 = 0.6543$; $Chi^2 = 3.74$, df = 2 (P = 0.1541); $I^2 = 46.5\%$					
**subgroup = 12 months**					
Sonneville 2023	7.00	4.4000	174	17.8%	7.00 [6.35; 7.65]
**subgroup = 18+ months**					
Azouvi 2016	7.20	4.5000	118	16.8%	7.20 [6.39; 8.01]
**Total (95% CI)**			**885**	**100.0%**	**6.70 [5.87; 7.52]**
**Prediction interval**					**[4.17; 9.22]**

Heterogeneity: $Tau^2 = 0.8877$; $Chi^2 = 32.89$, df = 6 ($P < 0.0001$); $I^2 = 81.8\%$
Test for subgroup differences: $Chi^2 = 2.11$, df = 3 (P = 0.5491)

Mean IV, Random, 95% CI

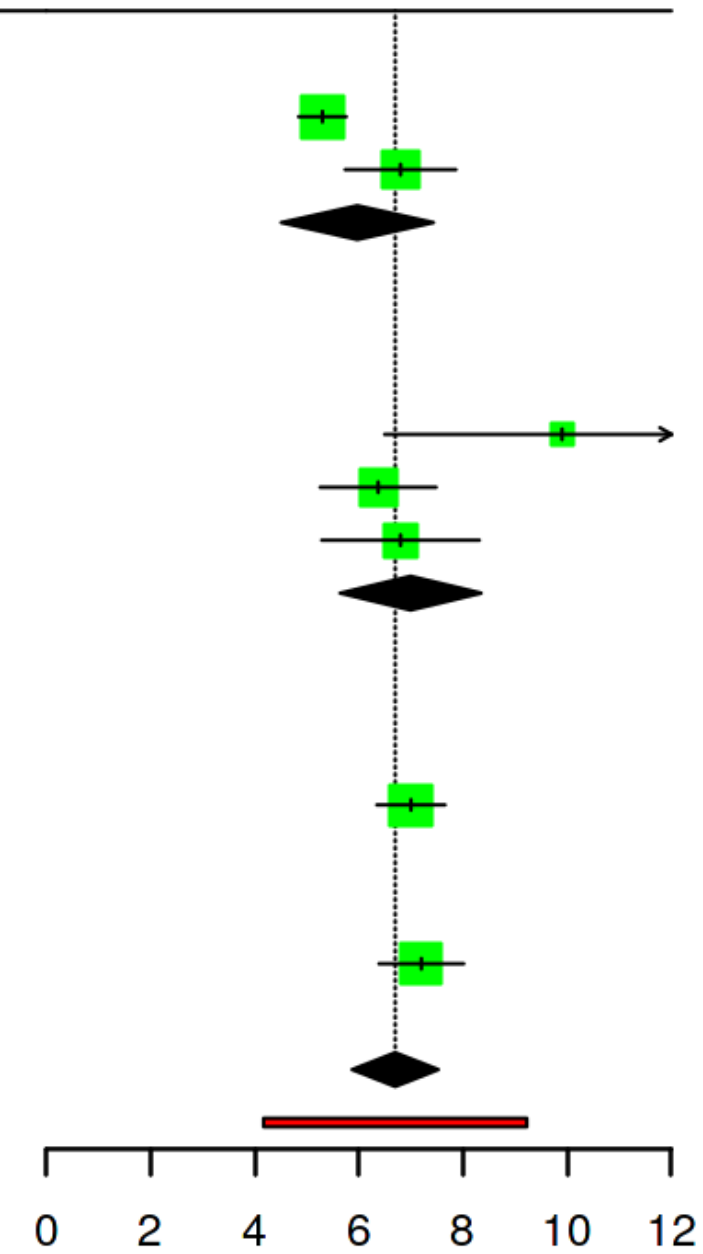

Footnotes:

[1] Seven studies analyzed totaling 885 subjects.
[2] Based on the analysis performed using random effects model with inverse variance method, the summarized raw means (MRAW) is 6.7 with a 95% confidence interval of 5.87-7.52.
[3] Significant heterogeneity detected ($p<0.01$), suggesting inconsistent effects in magnitude and/or direction. The I^2 value indicates that 82% of the variability among studies arises from heterogeneity rather than random chance.
[4] Only last timepoint data was included from studies with multiple timepoints of follow-up. Data from all timepoints can be found in Table S4.

Supplemental Table S5: Characteristics of manuscripts reporting on post-traumatic outcomes					
**Citation**	**Instrument Used**	**Study *N* at Follow-Up**	**Follow-Up Timeline (Mo)**	**PTS Prevalence (%)**	**PTS Score Mean (SD)**
Mikolić 2021	PCL-5	2054	6	9.6	NR
Presciutti 2021	PCL-5	70	3	NR	30.3
Bannon 2022	PCL-C	58<br>53<br>49	0<br>1.5<br>3	NR	35.4 (13.9)<br>30.5 (11.5)<br>35.0 (13.1)
Bannon 2020	PCL-C	16	0<br>1.5<br>3	NR	37.3 (7.8)<br>43.8 (10.6)<br>38.9 (11.8)
Cuthbertson 2004	DTS	78	3	14.1	8 [b]
Wintermann 2023	ASDS<br>-----------<br>PTSS-10 [a]<br><br>SCID-I	197	1<br>-----------<br>3<br><br>6	13.2<br>-----------<br>15.6<br><br>23.5	19 (19,19) [b]<br>-----------<br>22.0 (14.5-28.5) / 18.5 (13.0-27.0) [c]<br><br>24.0 (15.5-34.5) / 20.0 (14.0, 32.0) [c]
Diaz 2012	SCID-I	33	0	3.0	NR

Abbreviations: *ASDS*: Acute Stress Disorder Scale; *DTS*: Distress Tolerance Scale; *NR*: not reported; *PCL-5*: PTSD Checklist for DSM-5; *PCL-C*: PTSD Checklist for DSM-4; *PTS:* Post-traumatic symptomatology; *PTSS-10*: Post-Traumatic Stress Symptom Scale-10; *SCID-I*: Structured clinical interview

[a] PTSS-10 and SCID-I were used at t=3mo and t=6mo to identify participants with syndromal or subsyndromal post-traumatic stress disorder.

[b] Median (IQR when available) presented.

[c] Median (IQR) scores presented for mental health specialty consulted / not consulted for each follow-up time point.

Supplemental Figure S8:

Figure S8a:

Forest Plot: Post-Traumatic Stress Prevalence at ≤3 and ≥6 Months

Study or Subgroup	Events	Total	Weight	IV, Random, 95% CI
**Subgroup = 0-3 months**				
Diaz (instrument = SCID-I) 2012	1	33	3.6%	0.03 [0.00; 0.16]
Wintermann (instrument = ASDS) 2023	26	197	19.9%	0.13 [0.09; 0.19]
Cuthbertson (instrument = DTS) 2004	11	78	15.5%	0.14 [0.07; 0.24]
Wintermann (instrument = PTSS-10) 2023	17	112	17.9%	0.15 [0.09; 0.23]
**Total (95% CI)**		**420**	**56.9%**	**0.14 [0.09; 0.20]**
Heterogeneity: $Tau^2 = 0$; $Chi^2 = 2.82$, df = 3 (P = 0.4209); $I^2 = 0\%$				
**Subgroup = 6 months**				
Mikolić (instrument = PCL-5) 2021	197	2054	24.2%	0.10 [0.08; 0.11]
Wintermann (instrument = SCID-I) 2023	23	99	18.9%	0.23 [0.15; 0.33]
**Total (95% CI)**		**2153**	**43.1%**	**0.15 [0.00; 0.99]**
Heterogeneity: $Tau^2 = 0.5183$; $Chi^2 = 17.65$, df = 1 (P < 0.0001); $I^2 = 94.3\%$				
**Total (95% CI)**		**2573**	**100.0%**	**0.14 [0.08; 0.21]**
**Prediction interval**				**[0.04; 0.34]**

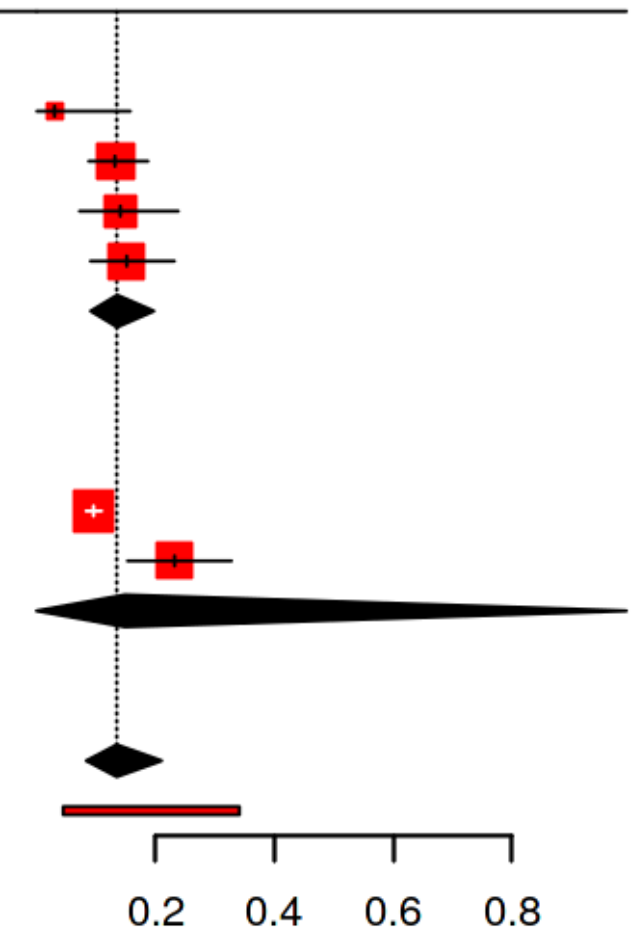

Heterogeneity: $Tau^2 = 0.1744$; $Chi^2 = 23.85$, df = 5 (P = 0.0002); $I^2 = 79.0\%$
Test for subgroup differences: $Chi^2 = 0.04$, df = 1 (P = 0.8430)

Footnotes:
[1] Six studies analyzed totaling 2,573 subjects. 5 different instruments used to measure post-traumatic stress across the studies.
[2] Based on the analysis performed using a random effects model with the inverse variance method and logit transformation, the summarized proportion is 0.14 with a 95% confidence interval of 0.08-0.21.
[3] Significant heterogeneity detected (*p*<0.01), suggesting inconsistent effects in magnitude and/or direction. The I^2 value indicates that 79% of the variability among studies arises from heterogeneity rather than random chance.

Figure S8b:

Forest Plot: Post-Traumatic Stress Prevalence at ≤6 Months

Study or Subgroup	Events	Total	Weight	IV, Random, 95% CI
**Subgroup = 0-6 months**				
Diaz (instrument = SCID-I) 2012	1	33	3.6%	0.03 [0.00; 0.16]
Wintermann (instrument = ASDS) 2023	26	197	19.9%	0.13 [0.09; 0.19]
Cuthbertson (instrument = DTS) 2004	11	78	15.5%	0.14 [0.07; 0.24]
Wintermann (instrument = PTSS-10) 2023	17	112	17.9%	0.15 [0.09; 0.23]
Mikolić (instrument = PCL-5) 2021	197	2054	24.2%	0.10 [0.08; 0.11]
Wintermann (instrument = SCID-I) 2023	23	99	18.9%	0.23 [0.15; 0.33]
**Total (95% CI)**		**2573**	**100.0%**	**0.14 [0.08; 0.21]**
Heterogeneity: $Tau^2 = 0.1744$; $Chi^2 = 23.85$, df = 5 (P = 0.0002); $I^2 = 79\%$				
**Total (95% CI)**		**2573**	**100.0%**	**0.14 [0.08; 0.21]**
**Prediction interval**				**[0.04; 0.34]**

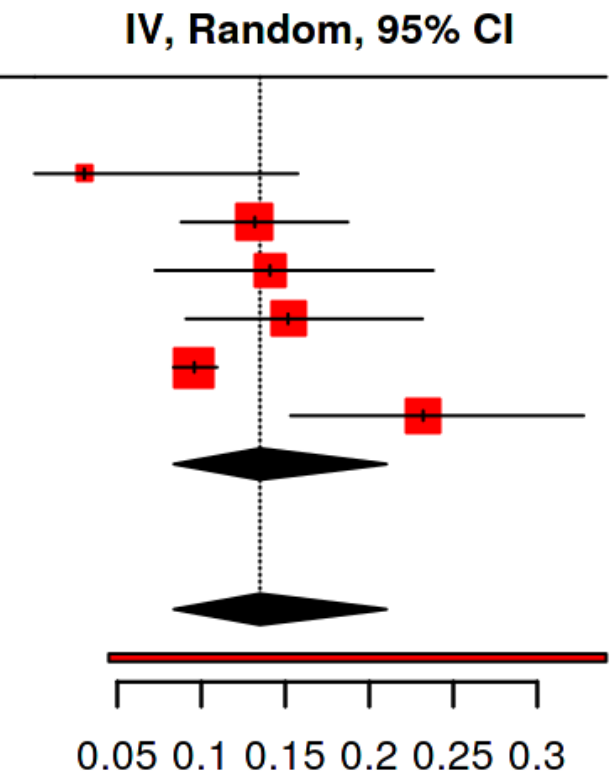

Heterogeneity: $Tau^2 = 0.1744$; $Chi^2 = 23.85$, df = 5 (P = 0.0002); $I^2 = 79.0\%$
Test for subgroup differences: $Chi^2 = 0.00$, df = 0 (P = NA)

Footnotes:

[1] Six studies analyzed totaling 2,573 subjects. 5 different instruments used to measure post-traumatic stress across studies.

[2] Based on the analysis performed using a random effects model with the inverse variance method and logit transformation, the summarized proportion is 0.14 with a 95% confidence interval of 0.08-0.21.

[3] Significant heterogeneity detected ($p<0.01$), suggesting inconsistent effects in magnitude and/or direction. The I^2 value indicates that 79% of the variability among studies arises from heterogeneity rather than random chance.

Supplemental Figure S9:

Forest Plot: Pooled PCL-C Scores for Post-Traumatic Stress Symptoms at 3 Months

Study or Subgroup	Mean	SD	Total	Weight	Mean IV, Random, 95% CI
**subgroup = 3 months**					
Bannon 2020	38.90	11.8000	16	32.9%	38.90 [33.12; 44.68]
Bannon 2022	35.00	13.1000	49	67.1%	35.00 [31.33; 38.67]
**Total (95% CI)**			**65**	**100.0%**	**36.28 [13.00; 59.57]**
Heterogeneity: $Tau^2 = 1.5026$; $Chi^2 = 1.25$, df = 1 (P = 0.2643); $I^2 = 19.8\%$					
**Total (95% CI)**			**65**	**100.0%**	**36.28 [13.00; 59.57]**
**Prediction interval**					**[8.27; 64.30]**

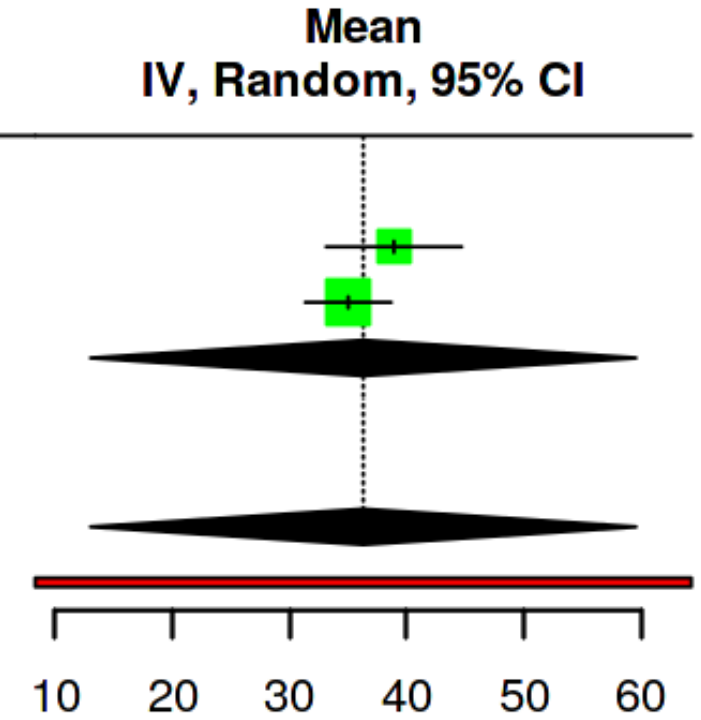

Heterogeneity: $Tau^2 = 1.5026$; $Chi^2 = 1.25$, df = 1 (P = 0.2643); $I^2 = 19.8\%$
Test for subgroup differences: $Chi^2 = 0.00$, df = 0 (P = NA)

Footnotes:
[1] Two studies analyzed totaling 65 subjects.
[2] Only last timepoint data was included from studies with multiple timepoints of follow-up. Data from all timepoints can be found in Table S5.
[3] Based on the analysis performed using random effects model with inverse variance method, the summarized raw means (MRAW) is 36.28 with a 95% confidence interval of 13.00-59.57.
[4] Significant heterogeneity not observed, indicating that effect sizes across studies are consistent in both magnitude and direction.

Supplemental Table S6: Characteristics in manuscripts reporting on general mental health outcomes					
**Citation**	**Instrument Used**	**Study *N* at Follow-Up**	**Follow-Up Timeline (Mo)**	**General Mental Health Prevalence (%)**	**General Mental Health Mean (SD)**
Meyers 2020 [a]	MOCS-A [b]	102	0	NR	31.9 (11.8)
Diaz 2012	BPRS [c]<br>-----------<br>SF-36-MH [d]	33	18	NR	7.1 (7.3)<br>-----------<br>67.2 (29)
Bründl 2021	ISR [e]	21	0<br>6	NR	0.9 (0.7)<br>0.7 (0.6)
Bannon 2020	GSE [f]	16	0<br>1.5<br>3	NR	28.8 (6.5)<br>26.8 (5.9)<br>28.4 (6.3)
Wintermann 2023	MSPSS [g]	197	1<br>3<br>6	NR	(---)<br>1.26 (0.79, 2.0) [j]<br>1.19 (0.75, 1.9) [j]
Costello 2024	Difficulties [h]	48	6	54	NR

Abbreviations: *BPRS*: Brief Psychiatric Rating Scale; *ISR*: ICD-10 Symptom Rating; *GSE*: General Self-Efficacy Scale; *MOCS-A*: Measure of Current Status, Part A; *MSPSS*: Multidimensional Scale of Perceived Social Support; *NR*: not reported; *SF-36-MH*: Mental health subscale of Short-Form Survey-36

[a] Multiple citations reporting on same population; all collapsed into single record in this table.
[b] The MOCS has two sections. Part A measures participants' ability to relax at will, recognize stress-inducing situations, restructure maladaptive thoughts, be assertive about needs, and choose appropriate coping responses as needed.
[c] Assesses the level of 18 symptom constructs such as hostility, suspiciousness, hallucination, and grandiosity.
[d] Data reported on 5-item mental health domain score of SF-36.
[e] A self-report questionnaire used to measure the severity of symptoms based on ICD-10 classification, including depression, anxiety, obsessive-compulsive disorder, somatoform disorders, and eating disorders.
[f] Assessed an individual's belief in their ability to cope with various challenging situations and adapt to change.
[g] 12-item measure of perceived adequacy of social support from three sources: family, friends, & significant other.
[h] Self-reported mental health difficulties.
[j] Raw MSPSS scores not provided; data represents odds ratio (95%CI).

<u>Supplemental Table S7:</u> Risk of Bias

Last Name	Representativeness of Exposed Cohort	Selection of non-exposed cohort.	Ascertainment of exposure(s)	Demonstration that PICS was not present at start	Comparability of exposed vs non-exposed cohort	Assessment of outcome	Follow-up enough for outcome to occur?	Adequacy of follow up
Mikolić	NR	N/A	NR	-	N/A	+	+	-
Meyers	+	N/A	+	-	N/A	+	+	-
Diaz	-	N/A	NR	+	N/A	+	+	-
Bannon 2022	NR	+	NR	+	++	+	+	-
Bründl	+	N/A	NR	-	N/A	+	+	+
Presciutti	-	N/A	+	-	N/A	+	+	-
Sadlonova	+	N/A	NR	+	N/A	+	+	+
Zhao	NR	+	+	-	-	+	+	+
Bannon 2020	+	N/A	+	+	N/A	+	+	-
Liao	-	N/A	+	+	N/A	-	+	NR
Azouvi	NR	N/A	+	-	N/A	+	+	-
Khajavikhan	+	N/A	+	-	N/A	+	+	+
Ragoschke-Schumm	+	N/A	+	-	N/A	+	+	-
Levine	-	N/A	+	+	N/A	+	+	+
Forslund	-	N/A	+	-	N/A	+	+	-
Geurts	NR	+	+	-	-	+	+	-
Demir	NR	-	+	-	-	+	+	NR
Cuthbertson	+	N/A	+	-	N/A	+	+	-
Quinn	+	N/A	+	-	N/A	+	+	+
Wintermann	NR	N/A	+	-	N/A	+	+	-
Sonneville	NR	N/A	+	-	N/A	+	+	+
Costello	-	N/A	+	-	N/A	-	+	+

Footnotes

[1] For each study, two independent reviewers assessed the risk of bias. Discrepancies were resolved through consensus.
[2] Randomized Controlled Trials were not evaluated differently during the risk of bias assessment.
